# Cu-engineered TiO_2_ thin films: defect-driven nonlinear optical responses for ultrafast frequency conversion and optical limiting

**DOI:** 10.1039/d6ra04163a

**Published:** 2026-07-15

**Authors:** Navyashree B, Ramseena Thundiyil, Junhui Lang, Katarzyna Ozga, Jaroslaw Jedryka, D. Guichaoua, S. Taboukhat, Saikat Chattopadhyay, Bhaghyesh A, Poornesh P, B. Sahraoui

**Affiliations:** a Manipal Institute of Technology, Manipal Academy of Higher Education Manipal Karnataka 576104 India poornesh.p@manipal.edu poorneshp@gmail.com; b Faculty of Electrical Engineering, Czestochowa University of Technology Armii Krajowej 17 PL-42-201 Czestochowa Poland; c Univ Angers, LPhiA, SFR MATRIX Angers F-49000 France; d Department of Physics, School of Physical and Biological Sciences, Manipal University Jaipur Rajasthan-303007 India

## Abstract

Cu-doped TiO_2_ thin films with varying Cu concentrations were fabricated by spray pyrolysis to investigate the influence of Cu incorporation on their nonlinear optical (NLO) properties. XRD analysis confirms the formation of anatase TiO_2_ with the emergence of Cu-related peaks at higher doping levels. XPS analysis verified the successful incorporation of Cu into the TiO_2_ matrix. *Z*-scan measurements under continuous-wave laser revealed an intensity-dependent transition from saturable absorption to reverse saturable absorption in the 1 and 2 wt% Cu–TiO_2_ films, with the 2 wt% film exhibiting the highest nonlinear absorption. Under nanosecond excitation, the 1 wt% Cu–TiO_2_ film displayed the strongest second-harmonic generation response, while the pristine TiO_2_ film showed the highest third-harmonic generation (THG) intensity. Maker fringe technique analysis under picosecond excitation demonstrated that the 2 wt% Cu–TiO_2_ film possesses the highest third-order nonlinear susceptibility (*χ*^(3)^ = 3.85 × 10^−21^ m^2^ V^−2^). Furthermore, the 1 and 2 wt% films exhibited superior THG performance under picosecond excitation, highlighting the role of ultrafast electronic nonlinearities. The enhanced NLO response is attributed to the synergistic effects of Cu-induced defect states, lattice distortion, and excitation-dependent nonlinear mechanisms. These findings establish Cu–TiO_2_ thin films as promising candidates for applications in optical limiting and ultrafast frequency conversion devices.

## Abstract 2

Tunable nonlinear absorption and enhanced harmonic generation in Cu-doped TiO_2_ thin films *via* Cu-induced defect engineering.

## Graphical abstract

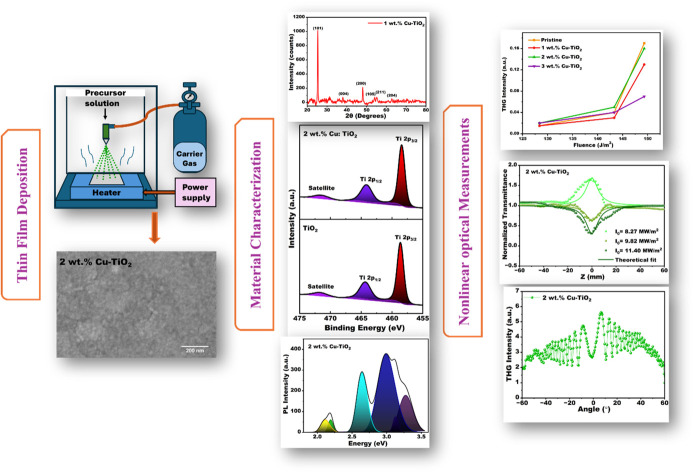

## 1 Introduction

Nonlinear optics (NLO) has turned out to be the most prominent field within the realm of photonics as it relies on materials whose parameters, such as refractive index and absorption coefficients, depend on the intensity of the incoming light.^1^ NLO properties offer the foundation on which a number of advanced optical technologies are built, such as ultrafast signal processing, frequency conversion, optical switching, and optical computing systems.^2,3^ The ideal materials are those that have large third-order nonlinearities, as they can be used to efficiently control light–matter interactions for the engineering of compact integrated photonic devices.^4,5^ There has been widespread research involving NLO materials, such as inorganic crystals,^6,7^ organic compounds,^8,9^ metal oxides,^10–12^ and polymers^13^ seeking variable systems that can support photonic applications. The nonlinear optical performance of a material is commonly assessed through parameters such as the nonlinear absorption coefficient (*β*), and nonlinear susceptibilities (*χ*^(2)^ and *χ*^(3)^). These parameters govern key phenomena including saturable absorption (SA), reverse saturable absorption (RSA), and harmonic generation processes. Materials exhibiting large nonlinear susceptibilities and efficient harmonic generation are highly desirable for applications such as optical limiting, all-optical switching, frequency conversion, and ultrafast photonic devices.^14,15^ Consequently, significant efforts have been devoted to engineering material composition and defect states to enhance these nonlinear parameters and tailor the optical response.^16^

In recent years, metal oxide semiconductors have drawn attention owing to their chemical stability, low cost, and applicability for practical uses. Titanium dioxide (TiO_2_) is preferred in this category. Anatase, brookite, and rutile are the three phases of TiO_2_.^17^ It is one of the widely studied materials because of its high photochemical stability, high refractive index, nontoxicity, and strong catalytic activity.^18,19^ TiO_2_ thin films are widely utilized in photocatalysis,^20^ photovoltaic devices,^21^ gas sensors,^22^ dielectric coatings,^23^ and emerging nonlinear optical applications.^24^

Yet the functional properties of TiO_2_ can be improved by optimizing its inherent properties. Doping has been a proven route toward such modifications, as the introduction of foreign ions into the TiO_2_ lattice can alter its electronic structure, create beneficial defect states, and influence crystallinity and charge carrier dynamics.^25,26^ Transition metal dopants, in particular, have shown considerable results in improving the functional performance of TiO_2_ based thin films.^27^ Copper (Cu) is a suitable dopant for TiO_2_ since its ionic radius being comparable to that of Ti^4+^, which permits substitution within the TiO_2_ lattice.^28^ Studies have reported that Cu doping can transform phase,^29^ tune the bandgap^30^ developing Cu–TiO_2_ a promising material system for advanced applications.

Various techniques have been employed for the fabrication of TiO_2_ thin films, including dip coating,^31^ spin coating,^32^ spray pyrolysis,^33^ chemical vapor deposition,^34^ and pulsed laser deposition.^35^ In this study, spray pyrolysis was opted as the deposition method because of its cost-effectiveness, simple apparatus and scalability.^36^ This technique permits one to have control over the composition of thin films, which can be achieved through the selection of solution concentration, spraying rate, and temperature of the substrate, which is particularly helpful when adding dopant ions into the TiO_2_ matrix. The spray pyrolysis technique has the ability to produce films with high adhesion without necessitating vacuum systems or complicated steps.

Many studies have been dedicated to the modulation of structural and optical properties of TiO_2_ by metal doping. Different fabrication methods and dopant species have been investigated to tailor microstructure and optical response. The following literature reports bring out the key advances in doped TiO_2_ nanostructures, particularly in terms of structure–property correlations relevant to optical and nonlinear optical applications. Farzaneh *et al.*^37^ published their research findings on the effect of doping with Al and Cu on TiO_2_ thin films synthesized by the sol–gel technique. Single-phase anatase TiO_2_ with no peaks related to dopants and their oxides was confirmed by the results of XRD analysis. Optical property analysis showed lower transmission and an enhanced absorption edge with doping with metals Al and Cu due to changes in the band structure. M. Rajabi *et al.*^38^ found an enhanced NLO response in La–TiO_2_ nanorod arrays when exposed to pulsed laser illumination. Nanorods were deposited on an FTO covered glass substrate. Single phase rutile TiO_2_ with lower lattice distortion was confirmed by the results provided by XRD analysis upon lanthanum doping. Notably, La doping led to a major improvement in the nonlinear absorption coefficient. T. Raguram *et al.*^39^ observed anatase phase of TiO_2_ when the concentration of metal copper was lower. However, when the Cu amount was maximized, XRD peaks related to copper oxides were found, accompanied by a reduction in average crystallite size. Raman spectroscopy further ensured Cu incorporation through the detection of Cu-related lattice vibrational modes at increased dopant concentrations. M.A. Khan *et al.*^40^ deposited Cu–TiO_2_ nanoparticles by doping Cu at various concentrations using the hydrothermal method. XRD analyses revealed that the prepared nanoparticles were of pure anatase structure, although the size of the crystallites increased with the addition of Cu. Furthermore, the PL analyses revealed that the PL intensity significantly lowered in the doped sample, thereby confirming the retardation in the recombination of charge carriers. O. Zakir *et al.*^41^ fabricated Ag, Fe, and Cu-doped TiO_2_ nanoparticles. XRD pattern of undoped TiO_2_ revealed a mixed anatase, rutile, and brookite phase, whereas all doped samples showed anatase structure with enhanced crystallinity and structural quality. Dopant incorporation resulted in reduced grain and crystallite sizes, indicating effective modification of the TiO_2_ lattice. F. Bensouici *et al.*^42^ examined Cu-doped TiO_2_ thin films and reported that all samples crystallized in the anatase phase; however, at higher Cu concentrations (8–10%), CuO phases emerged. Because CuO and Cu_2_O phases serve as sites where electron–hole pairs recombine, the efficiency of methylene blue degradation declined as the Cu^2+^ content increased. The role of Cu in the anatase to rutile phase transformation has been examined by Ciara Byrne *et al.*,^43^ who found that Cu doping enhanced the thermal stability of anatase, shifting the transition to higher temperatures. Also, XPS analysis revealed that increasing the temperature caused the partial reduction of Cu^2+^ to Cu^+^. These were supported by the work done by S. A. Ahmed,^44^ where he studied Cu–TiO_2_ nano powders and observed predominantly rutile-type crystallinity with minor anatase traces, along with an increase in lattice parameters and unit cell volume, attributed to Cu ions substitution into Ti lattice sites. V. Krishnakumar *et al.*^45^ reported that adding higher amounts of Cu reduced the crystallite size of TiO_2_ nanoparticles. FESEM observations also showed smaller particle sizes in Cu incorporated samples relative to the pristine. Additionally, the optical band gap narrowed upon Cu incorporation. Together, these works demonstrate that copper incorporation significantly modifies the microstructure, phase stability, and functional efficiency of TiO_2_.

Despite these advancements, the precise correlation between Cu concentration and the resulting nonlinear optical (NLO) properties in TiO_2_ thin films synthesized *via* spray pyrolysis remains largely unexplored. To address this gap, the present study focuses on the fabrication and systematic analysis of copper-doped TiO_2_ (Cu–TiO_2_) thin films with varying dopant levels. The objective is to elucidate how Cu incorporation modulates the structural, morphological, and linear optical characteristics, and how these changes dictate the NLO response. Uniquely, this work investigates nonlinear absorption (NLA) under continuous-wave (CW) excitation, while the second- and third-harmonic generation (SHG and THG) responses are analyzed in the nanosecond (ns) regime. Furthermore, picosecond (ps) laser excitation is employed to probe the angle-dependent THG response, providing insights into the ultrafast electronic contributions. Building upon our previous investigation into the effects of precursor concentration,^46^ this research aims to provide a comprehensive understanding of defect-engineered TiO_2_ nanostructures for advanced optoelectronic and photonic applications.

## 2 Experimental details

### 2.1 Synthesis of Cu–TiO_2_ thin films

**Fig. 1:**
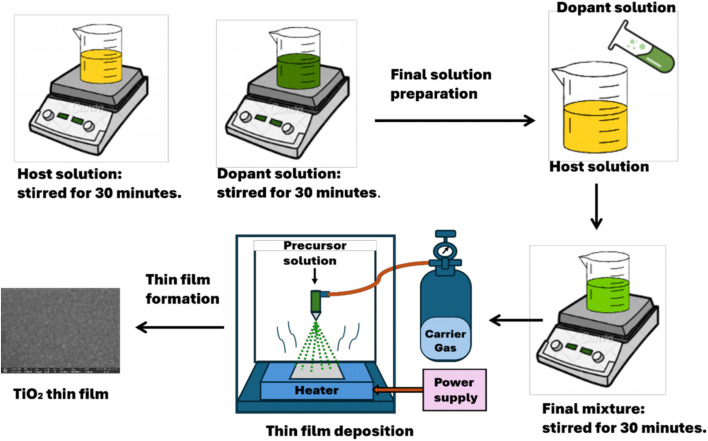
Schematic illustration of the thin film synthesis process.

**Fig. 2:**
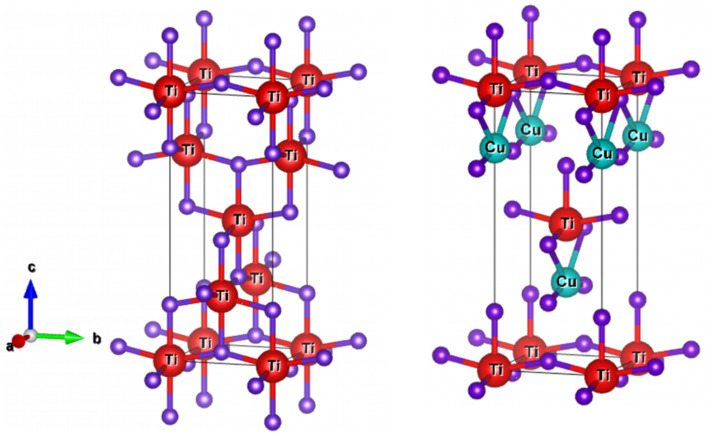
Structural models of pristine and Cu–TiO_2_.

In this study, copper-doped TiO_2_ (Cu–TiO_2_) thin films were deposited *via* the spray pyrolysis technique with varying dopant concentrations using a HOLMARC spray pyrolysis equipment (Model No: HO-TH-04). For the deposition of pristine films, a 0.2 M solution of titanium(iv) isopropoxide (Ti [OCH(CH_3_)_2_]_4_, Sigma-Aldrich) was prepared using ethanol (CH_3_CH_2_OH) as the solvent, and acetylacetone (CH_3_COCH_2_COCH_3_, Sigma-Aldrich) was added as a stabilizing agent. The dopant solution was obtained by dissolving copper (ii) chloride dihydrate (CuCl_2_·2H_2_O, MERCK) in ethanol to obtain a 0.2 M CuCl_2_ solution. Both the TiO_2_ precursor and the Cu dopant solutions were magnetically stirred for 30 min. With a speed of 2000 rpm at room temperature to ensure complete dissolution and homogeneity. Cu doping was performed by incorporating appropriate amounts of the dopant solution into the host solution to obtain final precursor solution with 1, 2, and 3 wt% Cu. The resulting mixtures were further stirred for an additional 30 min. To ensure uniform mixing. Prior to deposition, glass substrates were cleaned sequentially using soap solution, acetone, and isopropyl alcohol (IPA) in an ultrasonic cleaner to remove surface contaminants. The deposition was performed *via* spray pyrolysis by introducing the precursor solution at a controlled flow rate of 2 mL min^−1^. The thin films were coated onto the preheated substrates maintained at 400 °C under a working pressure of 0.5 bar. The thin films were synthesized following the same experimental parameters for all compositions. A flow diagram depicting the complete thin film synthesis route is provided in Fig. 1. The atomic arrangement in TiO_2_ and Cu–TiO_2_ crystals were visualized using the VESTA software. The obtained structural models are depicted in Fig. 2.

### 2.2 Material characterization

X-ray diffraction (XRD) experiments were conducted to assess the impact of Cu addition on the microstructural parameters of TiO_2_ using Rigaku SmartLab diffractometer operated at 100 mA and 45 kV with Cu Kα radiation (*λ* = 1.5406 Å) as source. The measurements were performed in Bragg–Brentano geometry with a step size of 0.05° and a scan rate of 2° min^−1^. Microstructural characteristics and defect-related features of the nanostructured Cu–TiO_2_ films were evaluated using Raman spectroscopy (Horiba LabRAM HR Evolution Raman spectrometer with an excitation source of 532 nm). Raman spectra were recorded using a neutral density filter with 10% transmission with an acquisition time of 10 s. Surface morphology was investigated using a field-emission scanning electron microscope (Apreo 2 SEM, Thermo Fisher Scientific) operated at an accelerating voltage of 2.0 kV and a working distance of 10 mm. Energy-dispersive X-ray spectroscopy (EDX) was employed for elemental composition analysis. The linear optical behavior of the Cu–TiO_2_ thin films was examined by employing a UV-vis spectrophotometer (Shimadzu UV-1900i). Absorbance and transmission spectra were acquired across the 190–1100 nm range to estimate the optical band gap and evaluate the films' absorption characteristics. The influence of Cu addition on the defect levels and radiative recombination in TiO_2_ matrix was evaluated by Photoluminescence (PL) spectrofluorometer (JASCO FP-8500) with an excitation source of 280 nm at room temperature. The oxidation states of the elements in the pristine and Cu–TiO_2_ thin films were analyzed by utilizing X-ray photoelectron spectroscopy (Thermo Scientific K-Alpha) with Al Kα X-ray source.

### 2.3 Nonlinear optical measurements

#### 2.3.1 *Z*-scan measurements

**Fig. 3:**
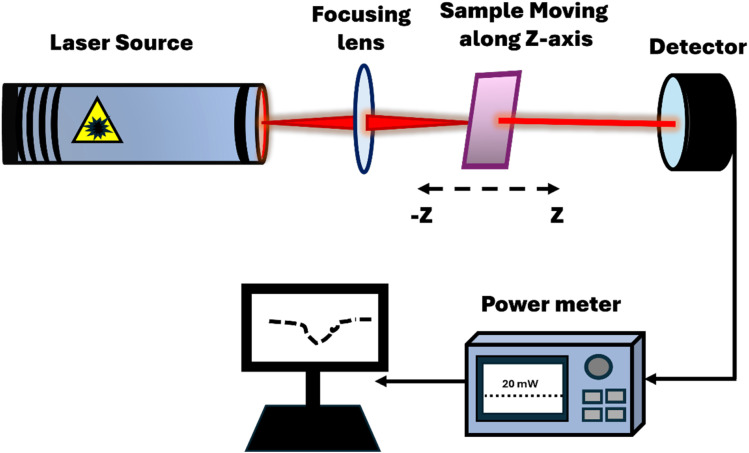
Schematic representation of *Z*-scan experiment.

The influence of addition of Cu on the NLO behavior of the Cu–TiO_2_ thin films was assessed by employing the open-aperture (OA) configuration of *Z*-scan method. The experiments were conducted on a system equipped with a translation stage (Thorlabs HRP350-EC-1) and a CW He–Ne laser (*λ* = 632.8 nm). To focus the beam on thin film, a convex lens (*f* = 5 cm) was used, and an aperture of 1.1 mm diameter was aligned in front of the lens. In order to adjust the beam power, neutral-density filters were applied. During data collection, the sample was programmed to move along the *Z*-axis, and the transmitted signal was captured using a photo detector linked to the data-logging unit of system. A schematic diagram of *Z*-scan experiment is presented in Fig. 3.

#### 2.3.2 Harmonic generations measurement

SHG and THG experiments were conducted to measure the harmonic generation in pristine and Cu–TiO_2_ thin films. The experimental arrangement incorporated ns laser source, reflective optics, a rotating stage, a silicon photo detector, a Glan–Taylor polarizer, a PMT, interference filters, an oscilloscope, and a computer-controlled data acquisition unit.

A nanosecond (ns) Nd: YAG laser (*λ* = 1064 nm) delivered radiation with a repetition rate of 10 Hz, pulse width of 8 ns, and an energy density close to 150 J m^−2^. Laser intensity and linear polarization were regulated by a Glan–Taylor polarizer positioned along the beam direction; its orientation was varied in 5° steps to achieve optimal THG signal detection at low input power. A semi-reflective mirror divided the incident beam, channeling one fraction to the silicon detector for timing reference while the remaining portion propagated toward the thin film.

**Fig. 4:**
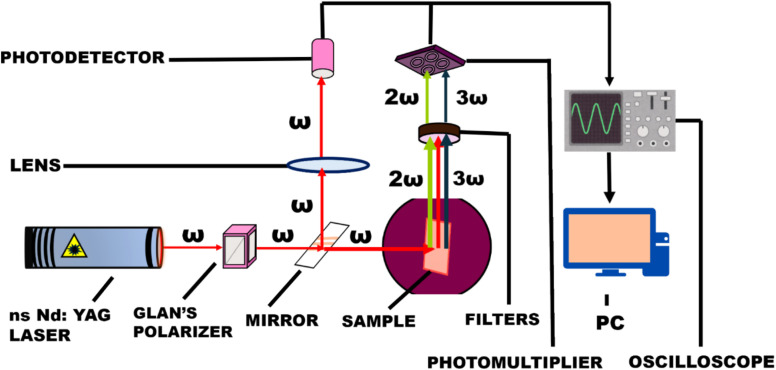
Schematic diagram of fluence dependent SHG and THG measurement.

Nonlinear interaction between the ns laser and the material produced harmonic emissions: the second harmonic, when generated, appeared around 532 nm, and the third harmonic emerged near 355 nm. These emitted wavelengths were isolated using appropriate interference filters and captured by the PMT, with detailed waveform acquisition accomplished on a Tektronix MSO 3054 oscilloscope. The entire optical system was enclosed within a dark, sealed chamber to suppress ambient light and guarantee accurate, noise-free SHG and THG measurements. A schematic diagram of harmonic generation experiment is presented in Fig. 4.

#### 2.3.3 Maker fringe technique

**Fig. 5:**
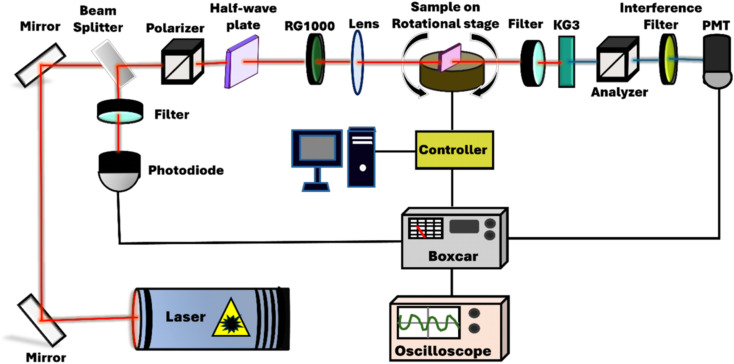
Schematic diagram of angle dependent THG measurement.

Maker fringe method was used to measure THG. The system consisted of a picosecond Nd:YVO_4_ laser (EKSPLA PL2250) which served as the excitation source with following parameters: 1064 nm wavelength, 30 ps pulse duration, 95 mJ energy, and a repetition rate of 10 Hz. The output beam was initially incident on a beam splitter, where a fraction of the beam was reflected toward a photodiode to monitor the signal. The transmitted beam was traversed through a polarizer followed by a half-wave plate, enabling precise control over the polarization and pulse energy. A lens was used to focus the polarized beam. The film was placed on a motorized rotational platform to enable accurate control of the incidence angle in the range of −60° to +60°, with a step size of 0.5°. The beam emerging after traversing through thin film was directed through a KG3 optical filter to suppress the fundamental radiation. Subsequently, the third harmonic component was selectively transmitted by using a filter (355 nm) and was detected using a photomultiplier. An oscilloscope, a controller and a computer were connected to the PMT and photodiode to ensure the time aligned operation, signal acquisition and data analysis. Thus, the resulting Maker fringe pattern of the THG signal was measured. Fig. 5 depicts the schematic diagram of Maker fringe experiment.

## 3 Results and discussion

### 3.1 XRD analysis

**Fig. 6:**
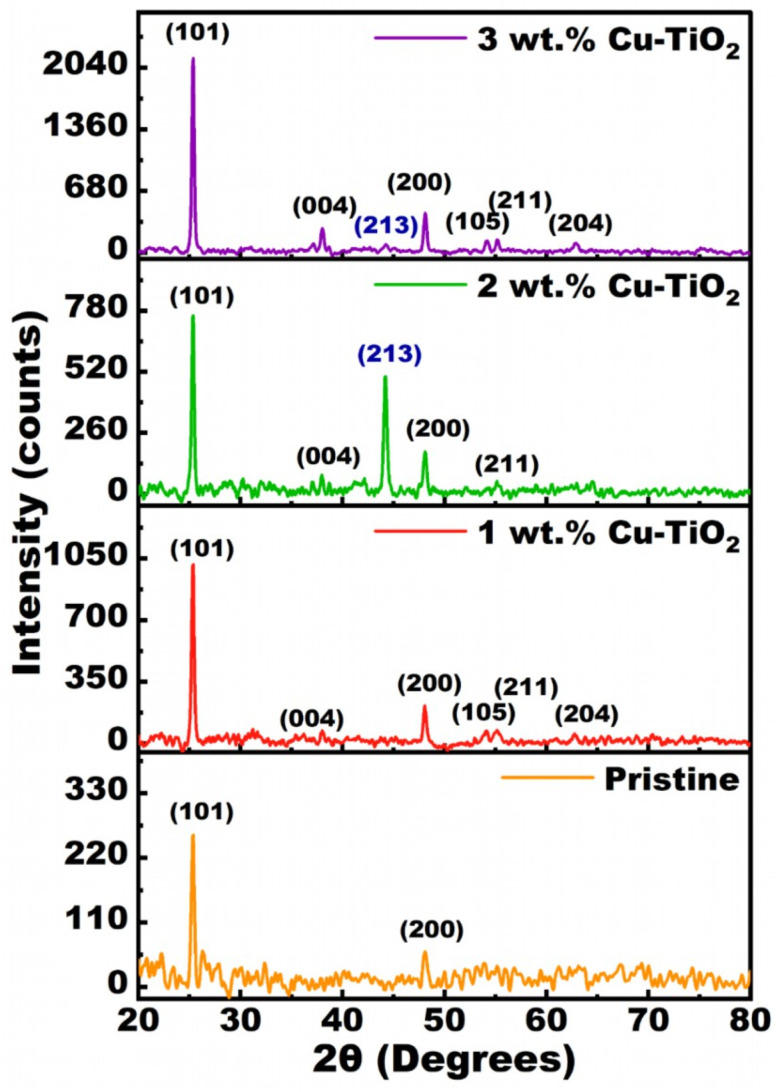
XRD pattern of pristine and Cu–TiO_2_ at different doping levels.

XRD analysis was conducted to assess the impact of Cu addition on the crystal structure, phase composition, and microcrystalline parameters of Cu–TiO_2_ thin films. Fig. 6 presents the XRD patterns of pristine and Cu–TiO_2_ thin films over a 2*θ* range of 20–80°. The XRD peaks are formed at 2*θ* ≈ 25°, 38°, 44°, 48°, 54°, 55°, and 62°. The dominant peak at 2*θ* ≈ 25° along (101) plane confirms the formation of tetragonal anatase TiO_2_, in agreement with JCPDS card no. 01-083-2243. In 2 wt% and 3 wt% Cu–TiO_2_ films, an additional peak at 2*θ* ≈ 44° corresponding to the (213) plane was observed, suggesting the occurrence of a secondary copper oxide phase in the doped thin films.^47–49^ However, 1 wt% Cu–TiO_2_ films did not exhibit any peaks related to copper, which confirms that lower Cu concentrations remain well incorporated into the host TiO_2_ lattice.

An enhancement in the intensity of the prominent peak along (101) is observed in Cu–TiO_2_ relative to the pristine, which reflects improvement in the crystalline structure with Cu doping.^50,51^ At 3 wt% Cu doping, crystallinity of TiO_2_ enhanced and a marginal reduction in the intensity of peak along (213) plane was observed. The crystallite size was estimated through Scherrer equation:^52^1
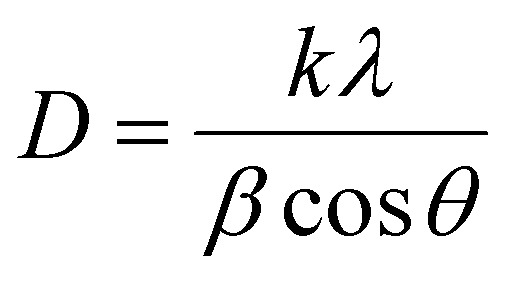
where *θ* is the Bragg's angle, *λ* = 0.15406 nm, *β* represents the FWHM of the TiO_2_ XRD peak, and *k* is Scherrer coefficient. The dislocation density (*δ*) was estimated using the relation:^53^2
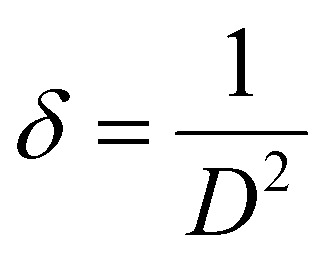


The strain (*ε*) in the films was determined using the formula:^54^3
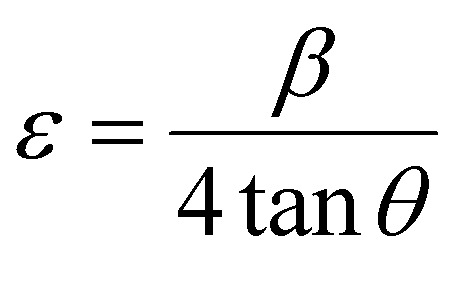


An increase in crystallite size was observed with increasing Cu doping concentration. This may be attributed to the successful incorporation of Cu ions into the TiO_2_ lattice, which can promote crystal growth and improve structural ordering during film formation, leading to a slight increase in crystallite size.^55^

The dislocation density decreases with increasing Cu dopant concentration. This behavior may be attributed to the successful incorporation of Cu ions into the TiO_2_ lattice, which promotes crystallite growth and improves atomic ordering, thereby reducing the density of lattice defects. Similarly, the lattice strain decreases with increasing Cu concentration. This reduction may be associated with improved structural relaxation and the effective accommodation of Cu ions within the TiO_2_ lattice, resulting in enhanced structural ordering and reduced lattice distortions.^56^

Lattice parameters and interplanar distance of films were estimated using the equation:^57^4
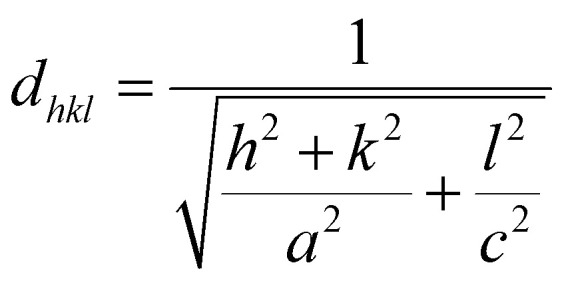
where *hkl* are miller indices.

A progressive lattice compression was observed with increasing Cu doping although Cu^2+^ (0.73 Å) has a larger ionic radius than Ti^4+^ (0.61 Å). This may be attributed to charge compensation through oxygen vacancy formation, which induces compressive strain in the TiO_2_ lattice.^58^ XPS results corroborate this by showing enhanced oxygen vacancy in 2 wt% Cu–TiO_2_. Table 1 presents the microstructural parameters of pristine and Cu–TiO_2_ thin films.

**Table 1:** Microstructural parameters of pristine and Cu–TiO_2_ thin films

Sample index	FWHM (deg.)	Crystallite size (nm)	Dislocation density (×10^14^ m^−2^)	Strain (×10^−3^)	Lattice parameter (Å)		Interplanar distance (Å)
					*a* = *b*	*c*	
Pristine	0.365	23.27	18.46	7.10	3.779	9.553	3.514
1 wt% Cu–TiO_2_	0.309	27.53	13.20	6.00	3.781	9.508	3.514
2 wt% Cu–TiO_2_	0.295	28.83	12.03	5.73	3.782	9.488	3.513
3 wt% Cu–TiO_2_	0.281	30.28	10.91	5.45	3.780	9.489	3.511

### 3.2 Raman analysis

**Fig. 7:**
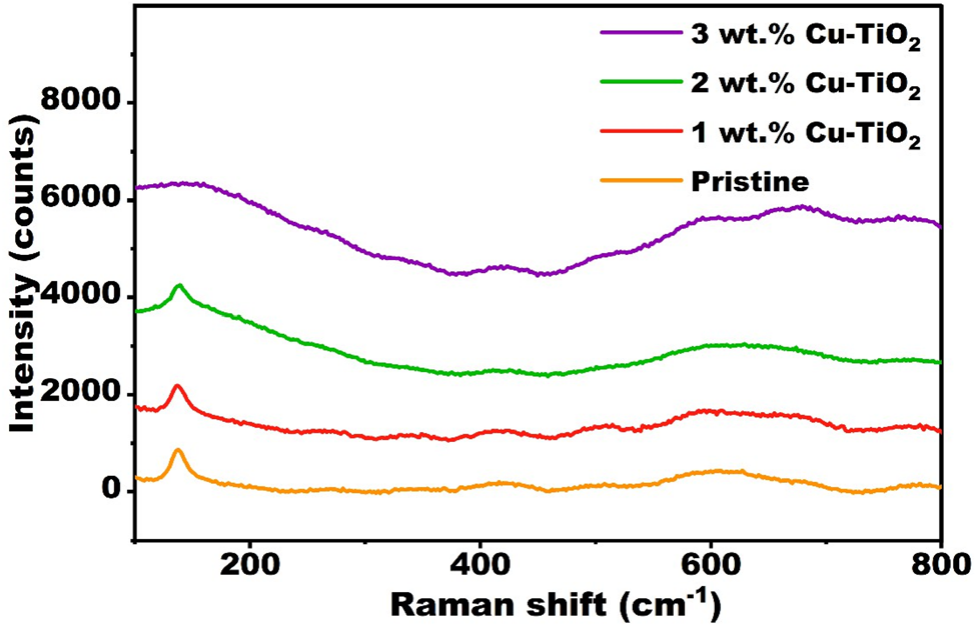
Raman spectra of pristine and Cu–TiO_2_ thin films.

The Raman study was performed to analyze the vibrational modes of the Cu–TiO_2_ thin films. Anatase TiO_2_ exhibits 15 optical phonon modes, represented as 1A_1g_ + 1A_2u_ + 2B_1g_ + 1B_2u_ + 3E_g_ + 2E_u_. Out of these, the E_g_, A_1g_, and B_1g_ are Raman active, while other modes are Raman inactive.^59^Fig. 7 depicts the Raman spectral profile of pristine and Cu–TiO_2_ films recorded over a range of 50–800 cm^−1^. Pristine Raman peaks were found at ∼138, 419, 513, and 607 cm^−1^. The peak at 138 cm^−1^ can be assigned to the E_g_ mode of anatase TiO_2_.^60^ The peak at 419 cm^−1^ can be assigned to B_1g_ Raman mode.^61^ Peak at 513 cm^−1^ is associated with A_1g_ + B_1g_ mode^62^ and the peak around 607 cm^−1^ is associated with the E_g_ mode.^63^ E_g_ mode originates from the symmetric stretching vibration of O–Ti–O. B_1g_ mode is associated with the symmetric bending vibration and A_1g_ mode corresponds to antisymmetric bending vibration of O–Ti–O bonds.^64,65^

The intensity of characteristic anatase Raman mode of 138 cm^−1^ gradually decreases as the Cu concentration increased up to 2 wt% Cu–TiO_2_. At 3 wt% Cu doping, the peak becomes undetectable, indicating that Cu-induced oxygen vacancies and the resulting local structural distortion in the lattice suppress the Raman-active E_g_ vibrational mode of anatase TiO_2_.^30^

E_g_ and A_1g_ + B_1g_ modes exhibit a slight shift towards higher wavenumber with Cu addition. Changes in these modes are commonly linked to inherent defects, such as oxygen vacancies.^66^ The addition of Cu induces oxygen vacancies in TiO_2_. The generation of these oxygen vacancies leads to lattice contraction, which in turn results in the peak shifting to higher wavenumber.^67^ In addition, the band near 607 cm^−1^ broadens with Cu concentration, and spans over a range of 625–730 cm^−1^ for 3 wt% Cu–TiO_2_. Such shift and broadening occur when dopant ions substitute at Ti^4+^ lattice sites, disrupting the original O–Ti–O bonds and forming Cu–O–Ti or Cu–O–Cu bonds, thereby affecting Raman active modes.^30^ Other than these peaks, other peaks appeared at ∼66 and 64 cm^−1^ for pristine and 1 wt% Cu–TiO_2_ are related to the Raman acoustic modes.^68^Table 2 presents the list of Raman modes of Cu–TiO_2_ thin films.

**Table 2:** Raman modes of pristine and Cu–TiO_2_ thin films

Sample index	Raman shift (cm^−1^)			
	E_g_	B_1g_	A_1g_ + B_1g_	E_g_
Pristine	138.08	419.51	513.03	607.21
1 wt% Cu–TiO_2_	139.11	418.40	513.52	639.19
2 wt% Cu–TiO_2_	139.57	418.95	—	634.47
3 wt% Cu–TiO_2_	—	418.09	521.31	680.09

### 3.3 FESEM analysis

**Fig. 8:**
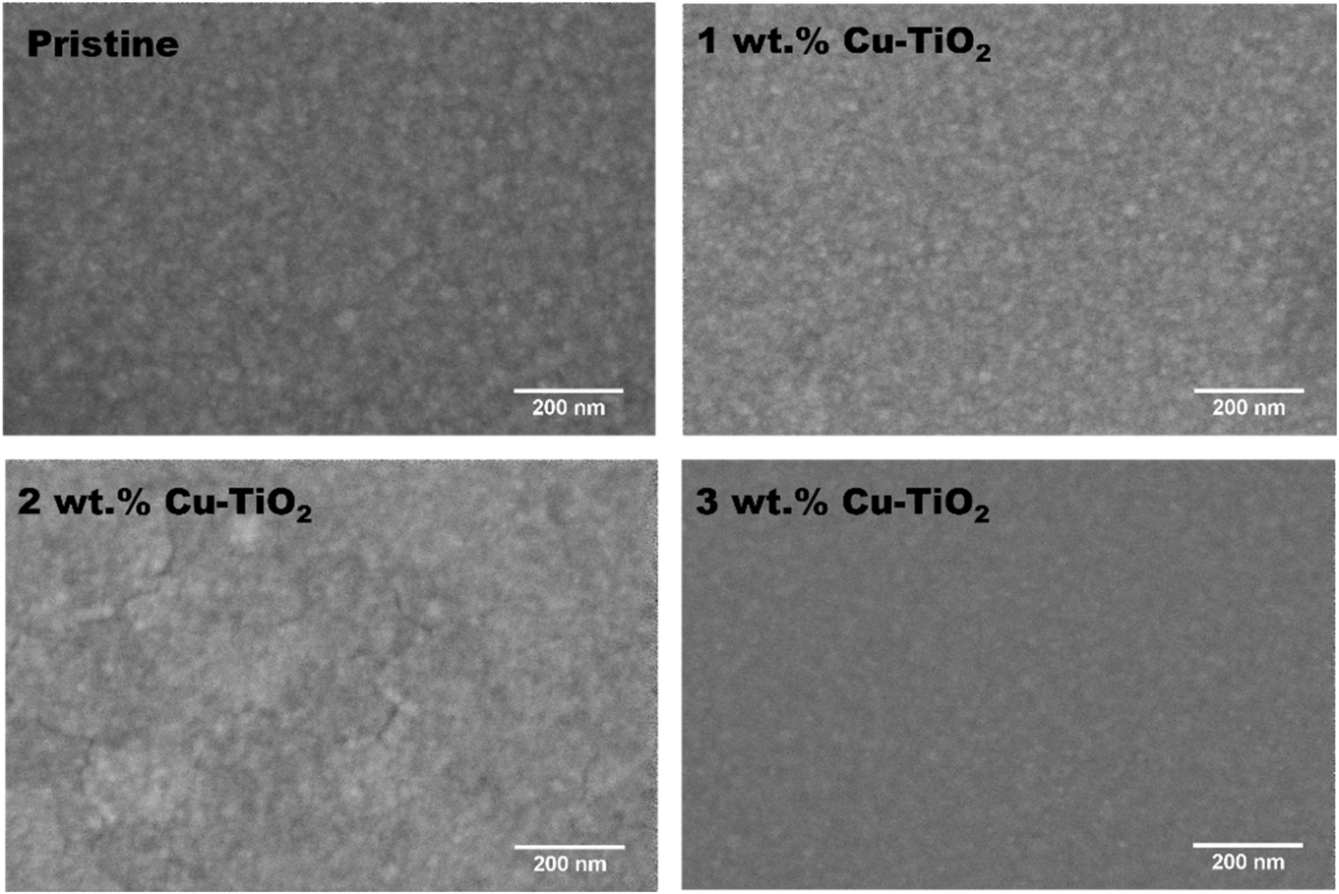
FESEM micrograph of pristine and Cu–TiO_2_ thin films.

The morphological features of Cu–TiO_2_ thin films was assessed by utilizing FESEM measurement. Micrographs recorded at high magnifications of 350 kX are presented in Fig. 8. The images reveal uniformly distributed nanoscale features without the presence of cracks, indicating the formation of dense and continuous films.

The surface exhibits closely packed grains with relatively smooth topography. Cu doping does not lead to major morphological distortion, and there is no segregated Cu rich clusters in the thin films.

**Fig. 9:**
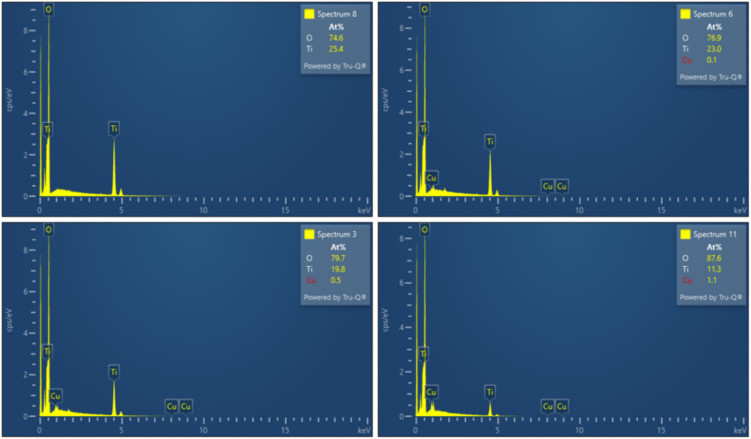
EDX spectra of pristine and Cu–TiO_2_ thin films.

EDX verifies the purity of elemental composition and successful doping of Cu into the TiO_2_ matrix. EDX spectra of pristine and Cu–TiO_2_ thin films are presented in Fig. 9. The undoped films show only Ti and O in near stoichiometric amounts, whereas the doped thin films clearly display the presence of Cu, with its concentration progressively increasing with the dopant level. This indicates dopant incorporation without detectable phase segregation. The gradual rise in Cu signal with higher doping corroborates the controlled substitutional addition of Cu into the TiO_2_ lattice.

### 3.4 UV-vis analysis

**Fig. 10:**
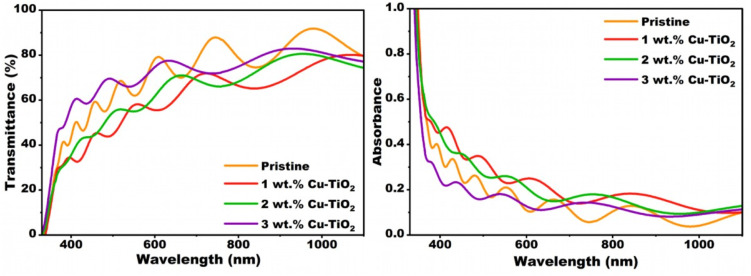
Optical transmittance and absorbance profiles of pristine and Cu–TiO_2_ thin films.

The linear optical properties of the pristine and Cu–TiO_2_ thin films were examined by UV-Visible characterization. Fig. 10 presents the optical transmission and absorption profiles of the Cu–TiO_2_ thin films at various dopant levels. The maximum transparency is exhibited by the pristine, and a reduction in transparency is observed in the Cu–TiO_2_ films. This decrease can be ascribed to enhanced light absorption and increased scattering induced by Cu related defects upon doping. The interference fringes observed in the transmittance and absorbance curves indicate that the deposited layers possess uniformity^42,69^ reflecting a smooth and consistent film surface.

The optical band gap of Cu–TiO_2_ films were calculated through Tauc's equation, given by:^70^

5 (*αhν*) = *A*(*hν* − *E*_g_)^*n*^

where *α* is absorption coefficient, *E*_g_ is optical bandgap, *hν* is the energy of incident photon, and *A* is the Tauc parameter, which is related to the degree of structural disorder within the material.^71^ And *n* takes the values of 
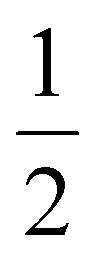
, 2, 3 and 
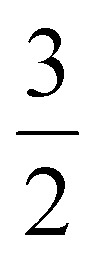
 for allowed direct, allowed indirect, forbidden direct, and forbidden indirect transitions, respectively.

**Fig. 11:**
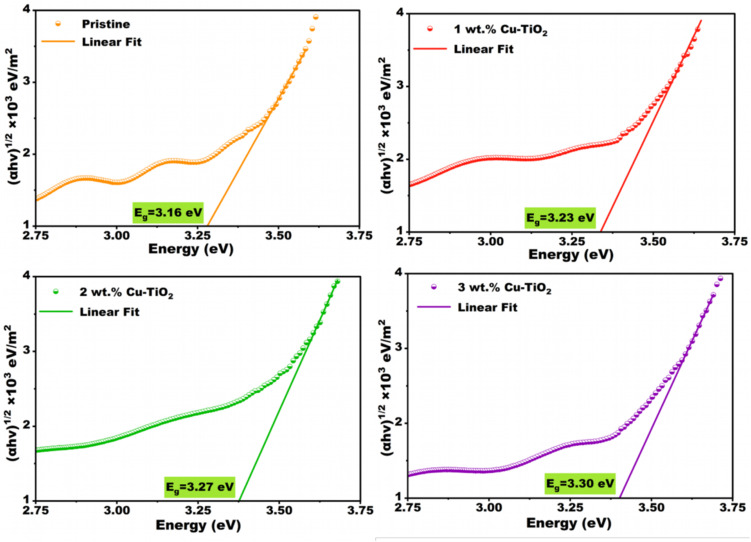
Tauc plots of pristine and Cu–TiO_2_ thin films.

Fig. 11 depicts the plot for indirect bandgap energies of pristine and Cu–TiO_2_ thin films.

The refractive index (*n*) can be estimated from the optical bandgap energy (*E*_g_) using empirical relations. The Moss relation was among the earliest proposed models correlating these two quantities, which is expressed as,^6^

6 *n*^4^*E*_g_ = 95 eV

In addition, Ravindra proposed a linear relationship between refractive index and optical bandgap energy, given by,^6^

7 *n* = 4.084–0.62 *E*_g_

The Herve and Van–Damme equation,^72^8
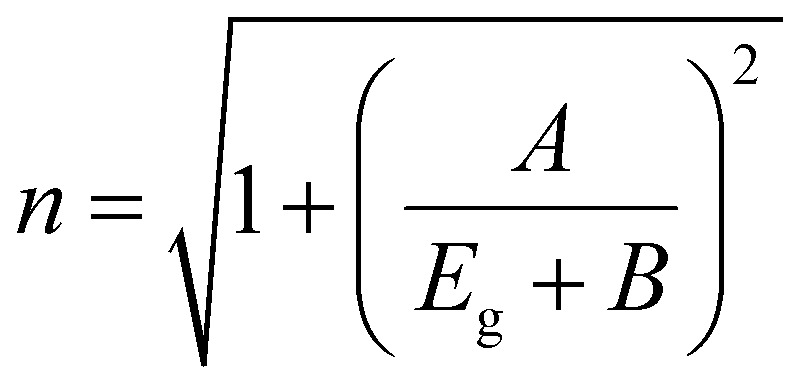
where *A* = 13.6 eV and *B* = 3.4 eV represent empirical constants, was ultimately adopted in the present work as it is based on classical oscillator theory and has been reported to provide better agreement with experimental refractive-index values than earlier empirical models.^8^

Pristine exhibits a bandgap energy of 3.26 eV. With an increment in Cu concentration the bandgap progressively widens to 3.40 eV at 3 wt% Cu–TiO_2_. This increase in bandgap energy is a result of the Burstein–Moss effect.^17,73^ When Cu is introduced into the TiO_2_ lattice, it can create donor-like states, which are electronic states located slightly below the conduction band that can donate electrons to the conduction band. As the concentration of these donor electrons increases, the lowest energy levels of the conduction band become progressively filled. Consequently, optical transitions from the valence band must occur to higher, unoccupied conduction-band states, effectively shifting the absorption edge toward higher energies, resulting in a blue shift in the bandgap energy. Refractive index was observed to decrease with an increment in Cu concentration. Overall, the opposite variation of refractive index with respect to bandgap is consistent with the Herve-Van Damme relation and reflects the interplay between impurity states and lattice restructuring upon Cu incorporation.

The thickness of the pristine and Cu–TiO_2_ thin films was determined using Parav software based on the Swanepoel envelope approach applied to the UV-vis transmission data. In this method, the interference peaks and valleys are selected manually, after which the software generates the upper and lower envelope curves and evaluates the optical path variation between successive fringes to estimate the film thickness.^74^ Film thickness decreased with Cu doping, likely due to Cu-induced modification of film growth kinetics, which reduced precursor utilization and altered nucleation and film growth behavior.

The extracted optical bandgap energies, corresponding refractive index values and film thickness for pristine and Cu–TiO_2_ films are summarized in Table 3.

**Table 3:** Optical bandgap energies and refractive index of pristine and Cu–TiO_2_ films

Sample index	Bandgap energy (eV)	Linear refractive index	Thickness (nm)
Pristine	3.26 ± 0.03	2.268	855.1
1 wt% Cu–TiO_2_	3.31 ± 0.02	2.254	657.3
2 wt% Cu–TiO_2_	3.38 ± 0.03	2.244	651.4
3 wt% Cu–TiO_2_	3.40 ± 0.02	2.236	636.2

### 3.5 PL analysis

**Fig. 12:**
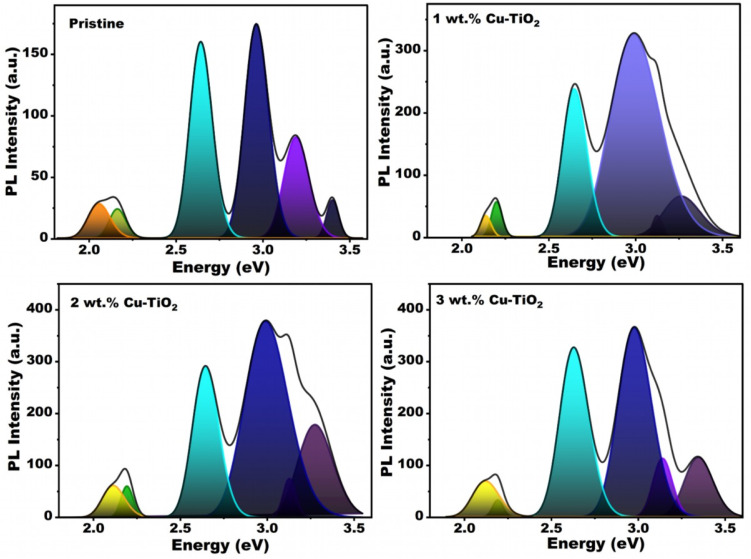
PL emission spectra of pristine and Cu–TiO_2_ thin films.

Photoluminescence measurements were conducted to study the defect states as well as radiative recombination mechanisms in pristine and Cu–TiO_2_ thin films, as PL is very sensitive to dopant induced changes in electronic structure. Gaussian deconvoluted PL profiles of the Cu–TiO_2_ films are depicted in Fig. 12, enabling the detection of individual peaks associated with defects. It is clear that Cu incorporation leads to an enhancement in the PL intensity of TiO_2_, indicating the enhancement of radiative recombination channels. The addition of Cu improves the defect states' generation such as oxygen vacancies and Ti^3+^ centers, which act as trap levels that facilitate radiative carrier recombination. The strengthened emission clearly demonstrates an enhanced defect assisted recombination. The list of the emission peaks, corresponding energies, and defect assignments for the undoped and Cu–TiO_2_ films are listed in Table 4.

**Table 4:** Summary of PL emission centers for pristine and Cu–TiO_2_ thin films, including emission energies, and the associated defect states

Sample index	Peaks	PL emission centers (eV)	Proposed defect states/mechanisms
Pristine	1	3.41	Intrinsic emission of anatase TiO_2_ thin film^75^
	2	3.20	Excitonic emission^76^
	3	2.97	Strong excitonic emission^77^
	4	2.64	Radiative recombination involving electrons at F/F^+^ or F^++^ states,^78^ oxygen vacancy related trap states^77,79,80^
	5	2.16	Oxygen vacancies^81^
	6	2.06	Defects of under coordinated Ti^3+^ ions^82^
1 wt% Cu–TiO_2_	1	3.26	Band edge emission^83^
	2	3.13	Near band edge emission^84^
	3	3.00	Strong excitonic emission^77^
	4	2.65	Radiative recombination from F/F^+^, or F^++^ defect centers^78^ oxygen vacancy related trap states^77,79,80^
	5	2.20	Radiative recombination involving electrons at F/F^+^ or F^++^ states^78^
	6	2.14	Additional electron states in the band gap^85^
2 wt% Cu–TiO_2_	1	3.28	Near band edge emission^83,86^
	2	3.13	Near band edge emission^84,86^
	3	3.00	Strong excitonic emission^77^
	4	2.64	Radiative recombination of electrons from F/F^+^ or F^++^ defect centers,^78^ oxygen vacancy related trap states^77,79,80^
	5	2.19	Radiative recombination from F/F^+^, or F^++^ defect centers^78^
	6	2.12	Additional electron states in the band gap^85^
3 wt% Cu–TiO_2_	1	3.35	Near band edge emission^86^
	2	3.14	Near band edge emission^84^
	3	2.98	Strong excitonic emission^77^
	4	2.63	Recombination from F/F^+^ or F^++^ states,^78^ oxygen vacancy related trap states^77,79,80^
	5	2.19	Recombination of electrons involving F/F^+^ or F^++^ states^78^
	6	2.12	Additional electron states in the band gap^85^

In the undoped TiO_2_, the high energy emissions observed around 3.41 eV results from intrinsic transitions of anatase TiO_2_ and peaks around 3.20 eV to 2.97 eV correspond to strong excitonic recombination processes. The broad blue emission originated at ∼2.64 eV is attributed to radiative recombination involving oxygen vacancy related defect levels, represented as F, F^+^, and F^++^ centers. These centers are related to oxygen vacancies with two, one, and zero trapped electrons, respectively,^64^ which introduce shallow donor levels below the conduction band. Electrons trapped in these sub-band gaps recombine with photogenerated holes, giving rise to the characteristic blue luminescence. In the longer wavelength regions, the visible emission at ∼2.16 eV band is associated with transitions from donor states of oxygen vacancies to acceptor levels. The emission peak at ∼2.06 eV is assigned to under coordinated Ti^3+^ sites, which form due to partial reduction of Ti^4+^ ions and act as deep level traps that facilitate radiative recombination.

Cu addition leads to changes in the emission pattern because of the Cu induced modifications of the local electronic structure of TiO_2_. There is an increased PL emission around ∼3.35 to 3.13 eV, which suggests enhanced band edge and excitonic recombination facilitated by electronic coupling between Cu states and the TiO_2_ lattice. The blue emission assigned to oxygen vacancies at ∼2.65–2.63 eV persists across all dopant concentrations, indicating that Cu incorporation does not hinder the creation of oxygen vacancies. The visible emissions in the 2.19–2.12 eV range are assigned to recombination through deeper level defect states. In particular, the rise of bands at ∼2.14 to 2.12 eV reflects the introduction of Cu induced electronic states within the band gap, which acts as intermediate recombination centers. These states are likely generated from Cu^2+^/Cu^+^ redox couples or Cu–O–Ti localized states, which influence charge localization and deepen trap mediated radiative pathways.

### 3.6 XPS analysis

**Fig. 13:**
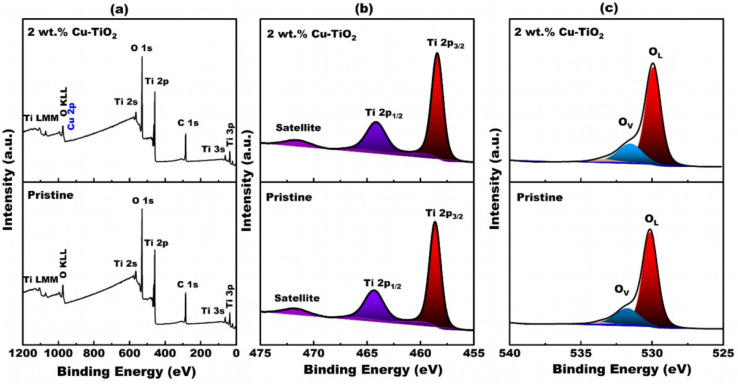
(a) XPS Survey spectra, core level spectra of (b) Ti 2p and (c) O 1s in pristine and 2 wt% Cu–TiO_2_ thin film.

The elemental composition of the pristine and 2 wt% Cu–TiO_2_ were examined by employing the XPS experiment. The XPS spectrum reveals signals corresponding to titanium and oxygen, along with a contribution from copper in the 2 wt% Cu–TiO_2_. The binding energy scale was calibrated by referencing the C 1s peak positioned at 284.8 eV. The survey spectra of pristine and 2 wt% Cu–TiO_2_ are presented in Fig. 13a.

#### 3.6.1 Core level Ti 2p spectra

The core level spectra of Ti 2p are presented in Fig. 13b. In pristine, Ti 2p core spectrum exhibited characteristic doublet peaks Ti 2p_3/2_ and Ti 2p_1/2_ at 458.63 eV 464.33 eV respectively, confirming the existence of Ti^4+^ in the anatase TiO_2_ lattice.^87,88^ In 2 wt% Cu–TiO_2_, these peaks shift slightly toward lower binding energies 458.43 and 464.13 eV, respectively. This shift towards lower Ti 2p_3/2_ and Ti 2p_1/2_ binding energies in Cu–TiO_2_ can be attributed to Cu^2+^ ion incorporation into TiO_2_ lattice. This changes electronic environment near Ti^4+^ ions, increasing oxygen vacancies.^89^ Despite the binding energy shift, the spin–orbit splitting (5.7 eV) remains unchanged in both pristine and 2 wt% Cu–TiO_2_ films, indicating the preservation of the Ti^4+^ state.^90^

#### 3.6.2 Core level O 1s spectra

The core level spectra of O 1s are presented in Fig. 13c. Two peaks were identified within O 1s core spectra: lattice oxygen (O_L_) and oxygen vacancies (V_O_). In pristine, the peak related to lattice oxygen appears at 530.13 eV, while the peak related to oxygen vacancies is found at 531.75 eV. For the 2 wt% Cu–TiO_2_, O_L_ is shifted to 529.92 eV, and the V_O_ peak is shifted to 531.52 eV. This shift along with shift in Ti 2p peak observed in 2 wt% Cu–TiO_2_ indicates that Cu incorporation changes the local structure and electronic states in TiO_2_ matrix.^89^ Additionally, a minor peak was observed at ∼533 eV in both pristine and 2 wt% Cu–TiO_2_ which corresponds to chemisorbed water species.^91^

The oxygen vacancy concentration was calculated using the 
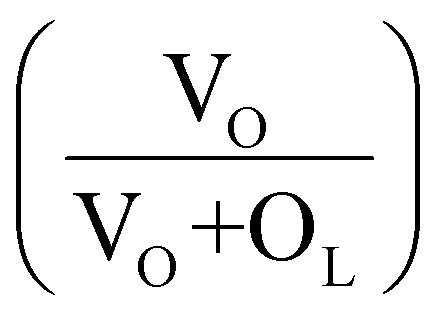
 area ratio. The area ratio increases from 21.6% in pristine to 23.9% in 2 wt% Cu–TiO_2_. The enhancement in oxygen vacancy is caused by the ionic radii mismatch between Ti and Cu, which induces lattice distortion and promotes oxygen vacancy formation.^92^

#### 3.6.3 Core level Cu 2p spectrum

**Fig. 14:**
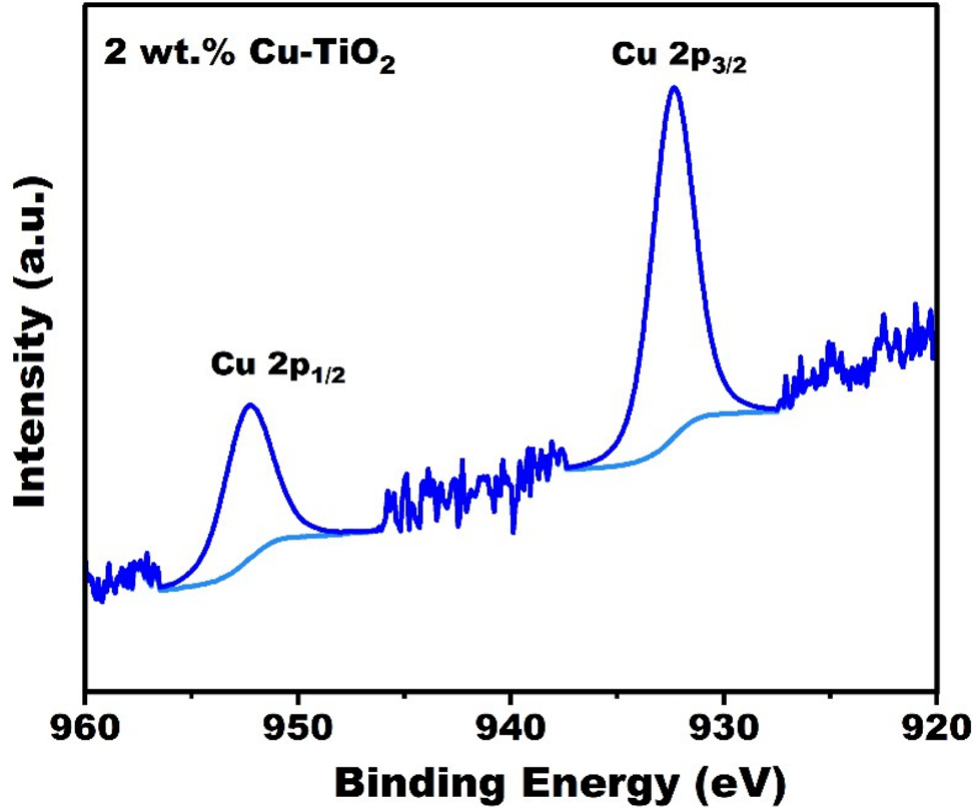
Core level spectrum of Cu 2p in 2 wt% Cu–TiO_2_ thin film.

The core level spectrum of Cu 2p is presented in Fig. 14, confirming the presence of Cu in the 2 wt% Cu–TiO_2_ film. In Cu 2p core spectrum, Cu 2p_3/2_ and Cu 2p_1/2_ peaks appear at 932.28 and 952.36 eV respectively, indicating the presence of Cu^2+^.^93,94^

Table 5 lists the summary of binding energies, FWHM, and peak areas obtained from the deconvolution of O 1s, Ti 2p and Cu 2p core level spectra of pristine and 2 wt% Cu–TiO_2_ thin films. The XPS results confirm Cu incorporation and reveal doping induced modifications to the chemical environment, including enhanced oxygen vacancy concentration and altered Ti–O electronic interactions.

**Table 5:** List of binding energies, FWHM and peak areas obtained from the deconvolution of Ti 2p, O 1s and Cu 2p spectra of pristine and 2 wt% Cu–TiO_2_ thin films

Sample index	Core level	Peak name	Peak position	FWHM	Area
Pristine	Ti 2p	Ti 2p_3/2_	458.63	1.25	346 889.76
		Ti 2p_1/2_	464.33	2.11	164 735.65
	O 1s	O_L_	530.13	1.23	391 454.70
		V_O_	531.75	2.00	108 214.64
		Chem. water	533.56	1.21	6933.10
2 wt% Cu–TiO_2_	Ti 2p	Ti 2p_3/2_	458.43	1.24	332 925.39
		Ti 2p_1/2_	464.13	2.13	158 643.85
	O 1s	O_L_	529.92	1.24	370 208.72
		V_O_	531.52	2.04	116 050.06
		Chem. water	533.34	1.76	13 764.81
	Cu 2p	Cu 2p_3/2_	932.28	2.31	10 735.76
		Cu 2p_1/2_	952.36	2.60	5311.22

### 3.7 Nonlinear optical studies

#### 3.7.1 *Z*-scan analysis

The NLA properties of Cu–TiO_2_ thin films were analyzed using the *Z*-scan technique.^95^ This single-beam experimental approach is a well-established and widely adopted method for characterizing NLO properties of materials.^1^ In this study, measurements were carried out in the OA configuration, in which the total transmitted laser beam is detected without any aperture filtering.^96^ Such a configuration directly probes the NLA response of the material. Typically, OA *Z*-scan measurements exhibit two characteristic absorption signatures: Saturable absorption (SA) takes place when absorption decreases with an increase in intensity, producing a transmission peak at focus, whereas reverse saturable absorption (RSA) results from intensity-enhanced absorption mechanisms, leading to a transmission dip at the focal position.^96,97^ The absorption coefficient as a function of intensity *I*, is expressed as^98^

9 *α*(*I*) = *α* + *β*_eff_

where *α* is the linear absorption coefficient, *β*_eff_ is the effective NLA coefficient. In an OA *Z*-scan experiment, the variation in normalized transmittance (*T*) with the position of sample (*z*) is obtained by curve-fitting using:^78,99^10
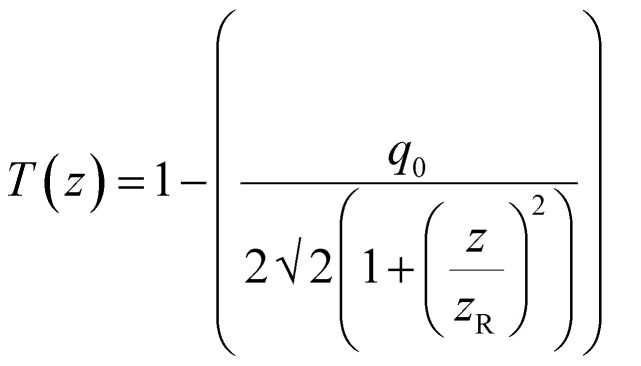
where *z*_R_ represents the Rayleigh length, *q*_0_ is the dimensionless NLA parameter, expressed as^99^

11 *q*_0_ = *β*_eff_*I*_0_*L*_eff_

where *L*_eff_ is the effective length and *I*_0_ represents the maximum intensity of the laser at focal point. Finally, the imaginary part of the third-order nonlinear susceptibility (Im*χ*^(3)^) is given by:^100^12
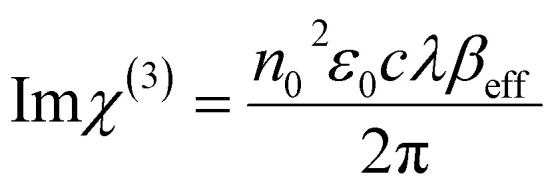
where *n*_0_ is the material's refractive index, *c* is the speed of light, and *ε*_0_ is the permittivity of free space.

**Fig. 15:**
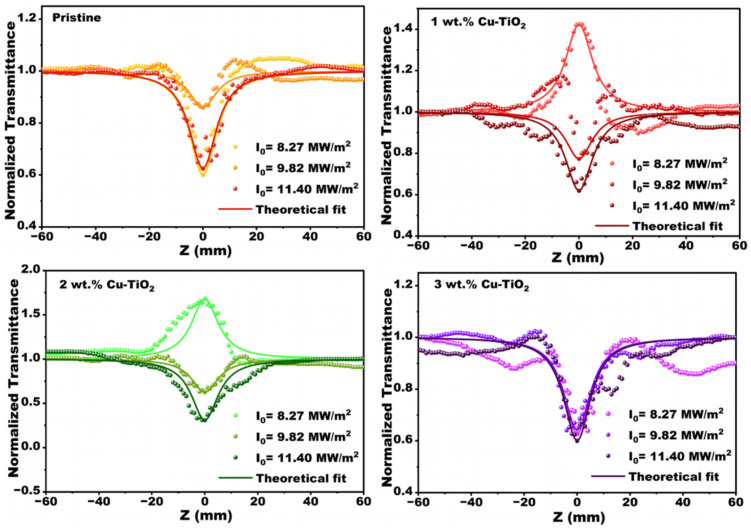
OA *Z*-scan curves of pristine and Cu–TiO_2_ thin films recorded at different incident laser intensities.

The OA *Z*-scan technique was employed to systematically investigate the NLA characteristics of pristine and Cu–TiO_2_ thin films under varying laser input intensities (8.27, 9.82, and 11.40 MW m^−2^), as shown in Fig. 15. The plain glass substrate exhibited a flat OA *Z*-scan trace with no variation around the focus confirming that it does not induce the NLO response of Cu–TiO_2_ films. Pristine and 3 wt% Cu–TiO_2_ thin films exhibit RSA nature, while 1 and 2 wt% Cu–TiO_2_ thin films exhibit both SA and RSA behavior. RSA may originate from mechanisms such as two photon absorption (TPA), multiphoton absorption (MPA), or excited state absorption (ESA).^101^

Pristine films exhibited only RSA behaviour which can be attributed to TPA process. In this study, the Cu–TiO_2_ thin films have bandgap energy of ∼3.3 eV, while the photon energy of the excitation source used is 1.96 eV. With such excitation, TPA can occur since the photon energy satisfies the criterion
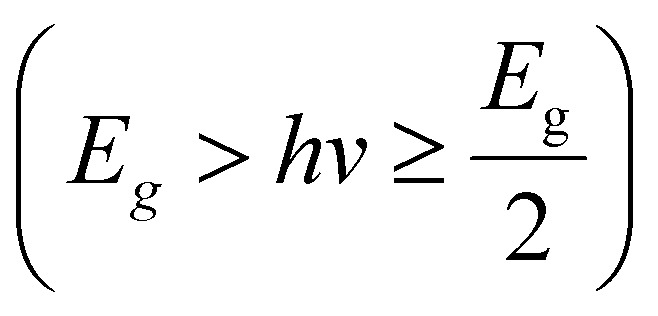
.^102^ So TPA is the primary NLA mechanism contributing to the observed NLO behavior of the Cu–TiO_2_ thin films. In TPA process, an electron in the valence band simultaneously absorbs two photons within a single optical cycle, enabling its transition to the conduction band through a virtual intermediate state.^103,104^ Alongside TPA, ESA may also contribute to the NLO response of the material, wherein charge carriers that are already promoted to excited or defect-related states undergo further photon absorption to reach higher energy levels, leading to an overall increase in absorption with increasing excitation intensity.^105^ In pristine, the highest *β*_eff_ value was observed at an incident intensity of 8.27 MW m^−2^, declined at 9.82 MW m^−2^ and increased at 11.40 MW m^−2^.

1 wt% Cu–TiO_2_ showed SA behavior at an incident intensity of 8.27 MW m^−2^. The observed SA behavior arises from the progressive depletion of ground-state carriers under low-intensity excitation, which reduces the probability of further photon absorption.^106^ With increasing laser intensity (9.82 and 11.40 MW m^−2^), a transition from SA to RSA was observed. This transition can be attributed to the competition between the ground state bleaching and thermally induced non-linearities. At higher intensities (11.40 MW m^−2^), the cumulative thermal effect, potentially coupled with ESA and TPA, dominates the optical response, leading to the observed RSA behaviour. *β*_eff_ value showed a progressive increment from −5.40 × 10^−1^ m W^−1^ at an intensity of 8.27 MW m^−2^ to 3.53 × 10^−1^ m W^−1^ at 11.40 MW m^−2^. This increment can be attributed to dominance of ESA mechanism induced by TPA process.^107^

Similarly, 2 wt% Cu–TiO_2_ showed SA behavior at an incident intensity of 8.27 MW m^−2^ and transitioned from SA to RSA with increasing laser intensity, showing a similar intensity-dependent switching mechanism. A significant enhancement in *β*_eff_ value was observed, increasing from −8.00 × 10^−1^ m W^−1^ at an excitation intensity of 8.27 MW m^−2^ to 5.90 × 10^−1^ m W^−1^ at 11.40 MW m^−2^, making the 2 wt% Cu–TiO_2_ exhibit the highest *β*_eff_ value among all the Cu–TiO_2_ thin films in this study.

However, the 3 wt% Cu–TiO_2_ film exhibited a stable RSA dominated response across all the excitation intensities and the *β*_eff_ value gradually decreased from 4.12 × 10^−1^ m W^−1^ to 3.22 × 10^−1^ m W^−1^ with increasing laser intensity. The calculated Im*χ*^(3)^ values follow a similar trend to that of *β*_eff_ values and are listed in Table 6.

**Table 6:** NLA coefficient and Im *χ*^(3)^ values of pristine and Cu–TiO_2_ thin films calculated for different laser intensities

Sample index	*I*_0_ (MW m^−2^)	*β*_eff_ (×10^−1^ m W^−1^)	Im *χ*^(3)^ (×10^−10^ m^2^ V^−2^)
Pristine	8.27	4.51	6.40
	9.82	1.33	1.89
	11.40	3.08	4.37
1 wt% Cu–TiO_2_	8.27	−5.40	−7.53
	9.82	2.43	3.38
	11.40	3.53	4.92
2 wt% Cu–TiO_2_	8.27	−8.00	−11.0
	9.82	3.70	5.10
	11.40	5.90	8.14
3 wt% Cu–TiO_2_	8.27	4.12	5.65
	9.82	3.28	4.49
	11.40	3.22	4.41

The highest value of Im*χ*^(3)^ value was observed for the 2 wt% Cu–TiO_2_. At an excitation intensity of 9.82 MW m^−2^, Im*χ*^(3)^ value increased from 1.89 ×10^−10^ m^2^ V^−2^ (pristine) to 5.10 ×10^−10^ m^2^ V^−2^ (2 wt% Cu–TiO_2_), representing a 2.7-fold increase relative to pristine. This enhancement can be attributed to optimal Cu incorporation, which introduces defect states and oxygen vacancies that modify the band structure and promote additional electronic transition pathways under laser excitation. However, 3 wt% Cu–TiO_2_ showed relatively lower Im*χ*^(3)^ values compared to the 2 wt% Cu–TiO_2_. This behavior may be attributed to dopant-induced defect clustering or increased non-radiative recombination, which can limit the effective population of excited states contributing to NLA. A comparison of the effective nonlinear absorption coefficient of the present Cu-doped TiO_2_ thin film with previously reported TiO_2_-derived materials is presented in Table 7.

**Table 7:** Comparison of nonlinear absorption coefficients (*β*_eff_) reported for TiO_2_-derived materials under different laser excitation conditions

Materials	Excitation source	*β*_eff_ (m W^−1^)	Ref.
CNT/TiO_2_–Pt composite	Q-switched Nd:YAG nanosecond laser with 4 ns pulse duration, and 10 Hz repetition rate	1.54 × 10^−6^	108
TiO_2_ thin film grown in an oxygen partial pressure of 16%	Continuous-wave He–Ne laser source operating at a wavelength 632.8 nm	−13.41 × 10^−2^	109
Bismuth-doped TiO_2_ colloids	Femtosecond laser with 150 fs pulse duration, and 76 MHz repetition rate	5.7 × 10^−9^	110
Gold-doped TiO_2_ colloids	Femtosecond laser with 150 fs pulse duration, and 76 MHz repetition rate	6.5 × 10^−9^	111
TiO_2_ nanoparticle colloids	Femtosecond laser with 100 fs pulse duration, and 80 MHz repetition rate	2.12 × 10^−11^	112
Cr-doped NiO nanopowder	Continuous-wave Nd: YAG laser operating at a wavelength of 532 nm	1.21 × 10^−2^	113
Mn-doped ZnO	Continuous-wave He–Ne laser source operating at a wavelength 633 nm	5.26 × 10^−2^	114
2 wt% Cu-doped TiO_2_ thin film	Continuous-wave He–Ne laser source operating at a wavelength 632.8 nm	5.90 × 10^−1^	Present work

#### 3.7.2 Fluence-dependent SHG under nanosecond laser

**Fig. 16:**
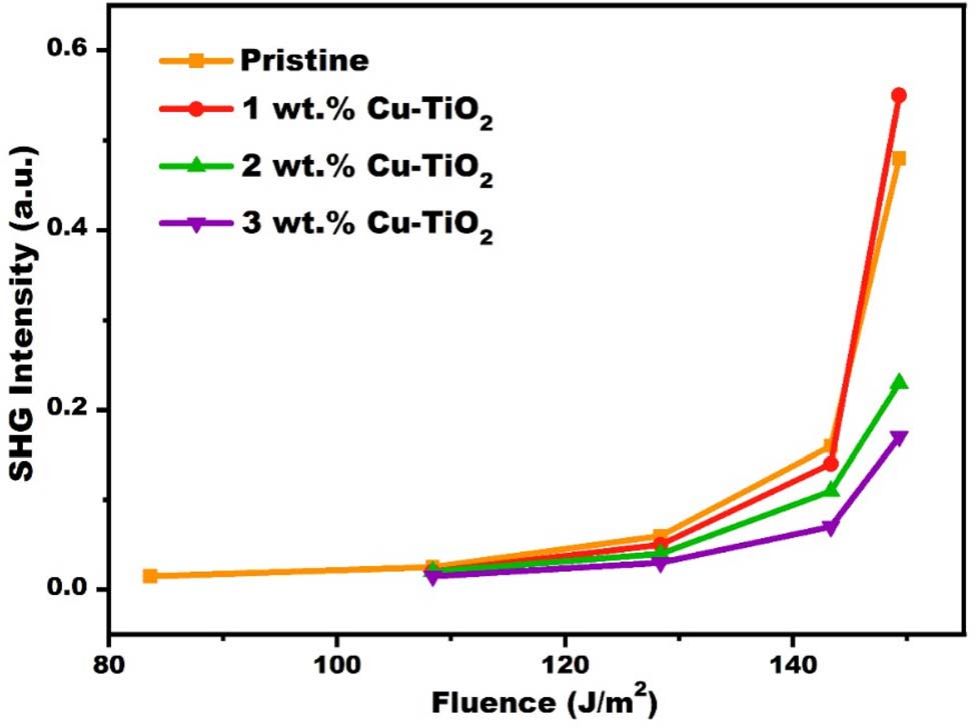
SHG intensity for pristine and Cu–TiO_2_ thin films.

SHG is a second-order NLO process in which two photons at a fundamental frequency interact within a nonlinear medium to generate a single photon at twice the frequency.^115^ SHG is allowed only in non-centrosymmetric materials, as centrosymmetric media possess inversion symmetry that cancels the second-order nonlinear response under the electric dipole approximation.^116^ Consequently, SHG is highly sensitive to crystal symmetry and structural order. At interfaces between materials or at surfaces, inversion symmetry is naturally broken, making reflection-based SHG highly sensitive to surface-induced non-centrosymmetry and interface-related defects.^117^ In this study, SHG measurements were performed in reflection mode using a nanosecond (ns) Nd: YAG laser (*λ* = 1064 nm) to assess the second-order NLO behavior and the associated electronic polarization behavior of the Cu–TiO_2_ thin films. The reflection geometry was specifically chosen to enhance sensitivity to surface-induced non-centrosymmetry and interface-related defects, as transmission-based SHG (Maker fringe) under picosecond excitation yielded negligible signals due to the bulk centrosymmetric nature of the films. This is consistent with the centrosymmetric crystal structure of TiO_2_, where the bulk second-order susceptibility *χ*^(2)^ vanishes by symmetry,^118^ and any local symmetry-breaking contributions from randomly oriented grains do not accumulate coherently along the transmission path due to the lack of net crystallographic texture in the polycrystalline films. In contrast, reflection-mode SHG captures the interface-localized response arising from surface/interface symmetry breaking.^117^ SHG experiments were carried out by varying the laser fluence ranging between 80–155 J m^−2^ as depicted in Fig. 16.

The SHG intensity showed minimal variation for all the thin films up to a fluence of 128 J m^−2^, followed by a sharp increase beyond 143 J m^−2^. The intensity of SHG signals initially increases with Cu doping, attaining a maximum value for the 1 wt% Cu–TiO_2_ film. This enhancement can be attributed to the introduction of an optimal density of Cu-related defects and oxygen vacancies. These defects induce local lattice distortions and break the centrosymmetric nature of the TiO_2_ matrix, thereby enhancing the effective second-order nonlinear polarization.^119^ However, with further increase in the dopant concentration SHG intensity decreases, becoming a minimum for the 3 wt% Cu–TiO_2_. This decline is likely due to excessive structural disorder and the potential formation of secondary phases that may restore a higher degree of centrosymmetry or increase scattering, thereby diminishing the coherent SHG response.

In addition to defect-induced distortions, the dense distribution of grain boundaries observed in FESEM contributes to local symmetry breaking at the film's surface, further facilitating the SHG process under reflection.

#### 3.7.3 Fluence-dependent THG under nanosecond laser

**Fig. 17:**
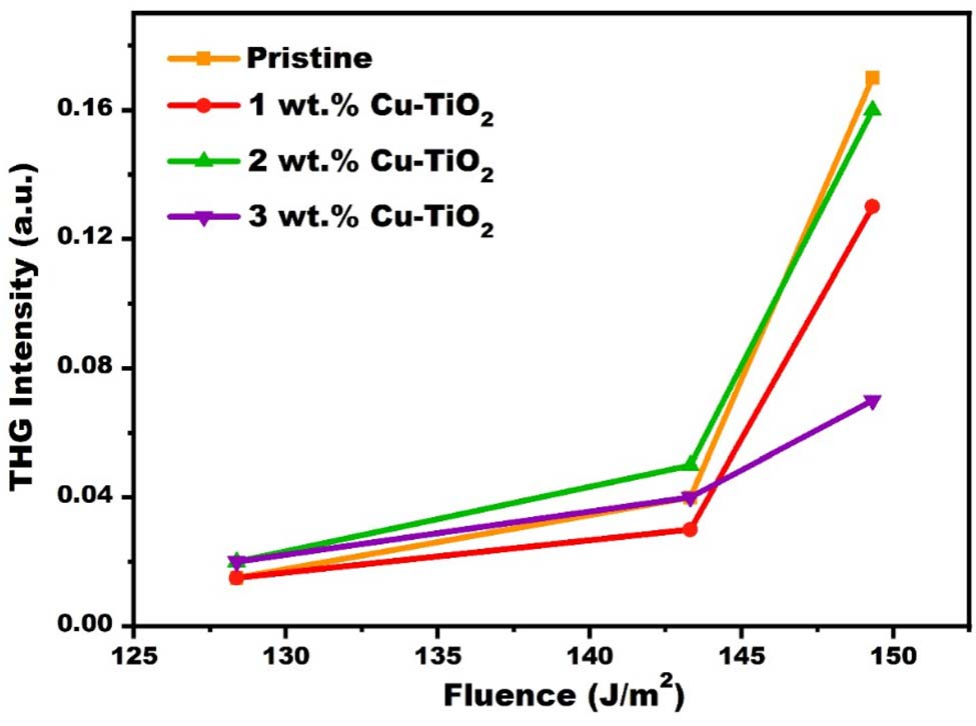
THG intensity for pristine and Cu–TiO_2_ thin films.

THG studies were performed to analyze the NLO response in Cu–TiO_2_ thin films. THG is a third-order nonlinear optical process arising from the interaction of three photons within a nonlinear medium to produce radiation at triple the fundamental frequency.^115^ THG is allowed in all materials irrespective of symmetry, since the third-order susceptibility (*χ*^(3)^) remains non-zero even in centrosymmetric media.^120^Fig. 17 depicts the dependence of THG intensity with the laser fluence in the range of 125–155 J m^−2^. The 2 wt% Cu–TiO_2_ film shows superior THG performance compared to pristine at lower fluences (130–147 J m^−2^). However, at higher fluences, the pristine thin film shows the highest THG intensity, surpassing the Cu-doped TiO_2_ films. The 3 wt% Cu–TiO_2_ film displays the lowest THG intensity overall. This crossover suggests that while Cu-doping enhances *χ*^(3)^ at low excitation levels, higher intensities may trigger excited-state quenching or saturation of the available electronic pathways in the doped matrix. The 3 wt% film consistently shows the lowest THG response, which can be attributed to the formation of impurity bands and increased structural disorder; these factors likely introduce significant non-radiative decay channels that suppress the coherent light–matter interaction required for efficient THG.

#### 3.7.4 Maker fringe technique analysis

**Fig. 18:**
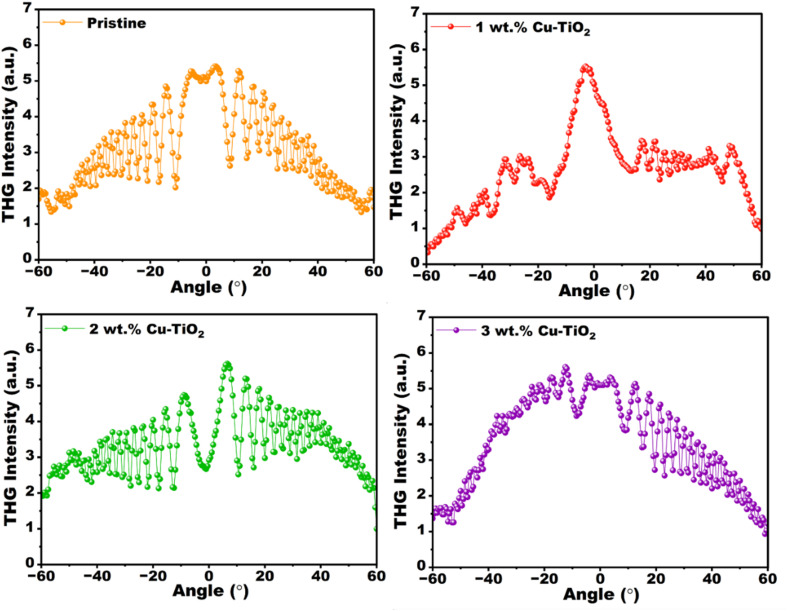
THG intensity of pristine and Cu–TiO_2_ thin films under *P*-polarization at an input laser energy of 129 µJ.

**Fig. 19:**
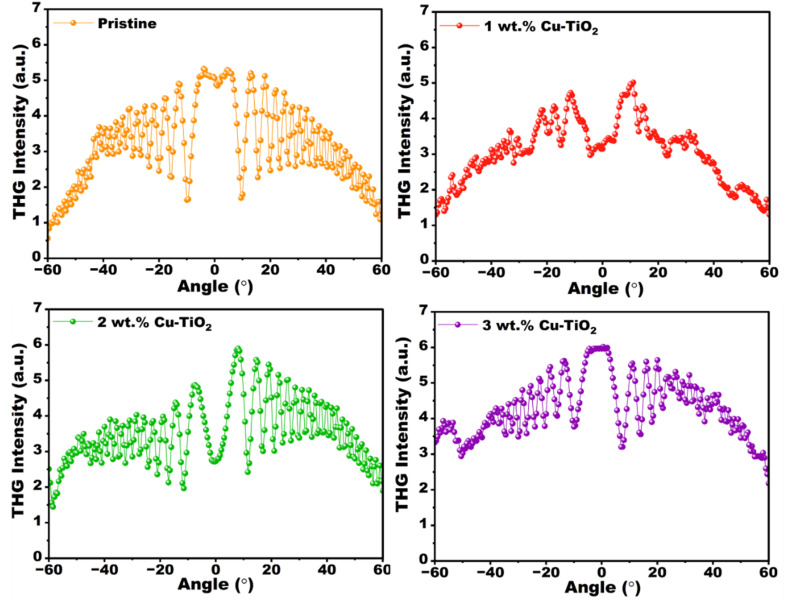
THG intensity of pristine and Cu–TiO_2_ thin films under *S*-polarization at an input laser energy of 129 µJ.

Maker fringe technique was employed using *P*- and *S*-polarized fundamental beams to study the impact of Cu introduction on the THG intensity of TiO_2_ thin films. Fig. 18 and 19 show dependence of intensity of THG signal with the incidence angle for Cu–TiO_2_ thin films under P and S polarization configurations, respectively. In both *P*- and *S*- configurations, the angular dependence of THG intensity exhibits a symmetric profile about normal incidence for the pristine film, indicating good film quality and accurate optical alignment.^121^ The Maker fringe patterns for the 2 wt% Cu–TiO_2_ film display a high degree of symmetry in both polarization configurations, indicating superior structural homogeneity and uniform film thickness. In contrast, the 1 wt% sample exhibits a less symmetric profile, which may be attributed to local variations in thickness or surface non-uniformity inherent to the spray pyrolysis process. To accurately determine the third-order nonlinear susceptibility (*χ*^(3)^), the linear absorption at the third-harmonic wavelength (355 nm) was taken into account. Since TiO_2_ possesses significant absorption in the UV range, the model proposed by K. Kubodera and H. Kobayashi model^122^ was employed. This approach allows for a precise estimation of *χ*^(3)^ by incorporating the absorption coefficient (*α*) and the integration of the nonlinear interaction length within the Cu–TiO_2_ thin films.^123^13
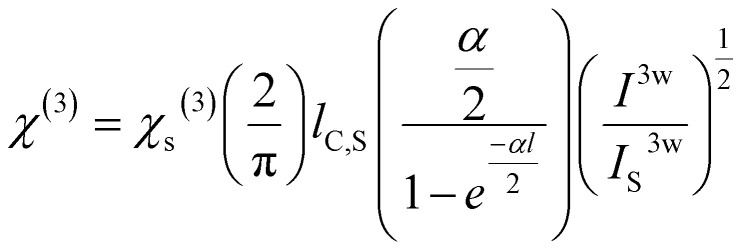
where *χ*_s_^(3)^ is the *χ*^(3)^ value of reference material (fused silica) with known value of 2.0 × 10^−22^ m^2^ V^−2^, *α* is the linear absorption coefficient at the third-harmonic wavelength, *l* is the thickness of sample and *l*_C,S_ is coherence length. *I*^3w^ and *I*_S_^3w^ represent the highest intensities of the THG signals exhibited by the Cu–TiO_2_ thin films and the reference respectively.

In *P*-polarization configuration, the maximum *χ*^(3)^ value was exhibited by 2 wt% Cu–TiO_2_, followed by pristine and then 1 wt% Cu–TiO_2_ and the lowest for 3 wt% Cu–TiO_2_. However, under *S*-polarization configuration *χ*^(3)^ values exhibit a different trend, with 1 wt% Cu–TiO_2_ showing the maximum *χ*^(3)^ response, followed by 2 wt% Cu–TiO_2_, pristine, and the lowest for 1 wt% Cu–TiO_2_. Notably, the variation in *χ*^(3)^ values in the case of *P*- and *S*- polarization configurations is very small. The difference in optimal Cu concentration under *P*- and *S*-polarization configurations may be attributed to variations in the intrinsic material properties.

The maximum *χ*^(3)^ value was exhibited by 2 wt% Cu–TiO_2_ can also be ascribed to the optimal Cu incorporation, which increases defect density such as oxygen vacancies and induces local lattice distortion, thereby enhancing the nonlinear optical properties. These defects modify the electronic band structure, providing intermediate states that enhance the nonlinear polarization *via* resonance effects. However, for the 3 wt% film, the *χ*^(3)^ values fall below even those of the pristine TiO_2_. This decline is likely due to excessive dopant-induced disorder and defect clustering, which disrupt the lattice periodicity and increase non-radiative recombination rates, thereby quenching the third-order nonlinear response. The obtained values of *χ*^(3)^ are listed in Table 8. Table 9 lists the reported *χ*^(3)^ values of various nanomaterials measured using the Maker fringe method under picosecond laser excitation. The variation in *χ*^(3)^ demonstrates that NLO behavior is strongly dependent on the material system and its microstructural and electronic characteristics.

**Table 8:** Estimated values of *α* and *χ*^(3)^ for Cu–TiO_2_ thin films

Sample index	*α* × 10^6^ (m^−1^)	*χ* ^(3)^ × 10^−21^ m^2^ V^−2^	
		*P*-polarization	*S*-polarization
Pristine	4.69	3.65	3.67
1 wt% Cu–TiO_2_	4.98	3.62	3.80
2 wt% Cu–TiO_2_	4.73	3.85	3.76
3 wt% Cu–TiO_2_	3.43	3.52	3.40

**Table 9:** Reported *χ*^(3)^ values of various nanomaterials

Materials	*χ* ^(3)^ × 10^−21^ m^2^ V^−2^	Ref.
2% Ni-doped CdS	5.40	124
NiO	1.94	125
MgO	4.89	126

#### 3.7.5 Input energy-dependent THG under picosecond laser

**Fig. 20:**
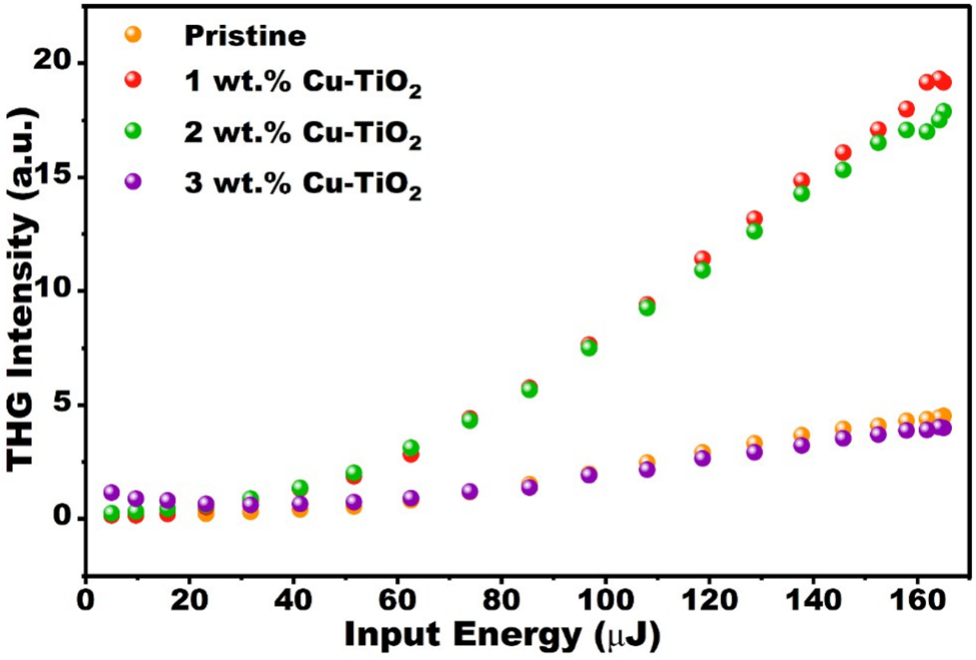
Dependence of THG intensity with input energy in pristine and Cu–TiO_2_ thin films.

The THG intensity of pristine and Cu–TiO_2_ thin films was further studied as a function of input laser energy using a picosecond Nd: YVO_4_ laser (*λ* = 1064 nm). The use of picosecond pulses is crucial here, as it allows the nonlinear response to be dominated by ultrafast electronic polarization, while minimizing contributions from slower thermal effects. The variation of THG intensity in Cu–TiO_2_ films against input energy in the range of 0–170 µJ is presented in Fig. 20. The pristine thin film exhibited a relatively weak THG signal. Upon Cu incorporation, a pronounced enhancement in THG response is observed. While the 1 wt% and 2 wt% Cu–TiO_2_ films show the highest intensities, their profiles are remarkably similar; these minor differences are not statistically significant and can be attributed to experimental uncertainties, such as slight laser drift over time. This suggests that a doping range between 1 and 2 wt% represents an optimal window where Cu-induced defect states and lattice distortions effectively increase the electronic polarizability of the TiO_2_ matrix. In contrast, the 3 wt% Cu–TiO_2_ film exhibits a marked decrease in THG intensity, even lower than that of pristine. At higher Cu concentrations, increased structural disorders^127^ can lead to enhanced carrier scattering and nonradiative recombination pathways. These effects suppress the coherent nonlinear polarization responsible for THG, resulting in a diminished third-order nonlinear response.

#### 3.7.6 Summary of NLO studies

In *Z*-scan technique under CW laser excitation, pristine and 3 wt% Cu–TiO_2_ exhibited RSA behaviour while 2 and 3 wt% Cu–TiO_2_ films showed a transition from SA to RSA with the increasing laser intensity. The 2 wt% Cu–TiO_2_ film showed the highest *β*_eff_ and Im*χ*^(3)^ values of 5.90 × 10^−1^ m W^−1^ and 8.14 × 10^−10^ m^2^ V^−2^, respectively. This is attributed to an optimal balance between defect density and crystallinity in the 2 wt% Cu–TiO_2_ film.

SHG measurements under ns laser excitation in reflection mode, with varying fluence showed maximum response for the 1 wt% Cu–TiO_2_ film, arising from Cu-induced defects that introduce local lattice distortions and effectively break the centrosymmetric nature of the TiO_2_ matrix. THG measurements under ns laser excitation in reflection mode, with varying fluence, showed maximum THG response for the pristine film.

The Maker fringe analysis under ps laser excitation further confirms that the 2 wt% Cu–TiO_2_ film possesses the highest *χ*^(3)^ value of 3.85 × 10^−21^ m^2^ V^−2^, consistent with optimal Cu incorporation. Furthermore, THG studies with varying input energy show that the 1 wt% and 2 wt% films yield the strongest and most comparable THG responses. The variation in THG intensity under ns and ps-excitation is fundamentally governed by pulse duration-dependent nonlinear interaction and their sensitivity to defect states. Under ps excitation, the interaction is restricted to ultrafast timescales, effectively suppressing thermal and cumulative contributions and isolating the intrinsic electronic response of the material.^128^ In this regime, the 1 and 2 wt% Cu doping range is optimal, as mid-gap levels and oxygen vacancies enhance electronic polarizability. Under ns excitation, the pristine film exhibits the highest THG response, as the longer pulse duration enables significant carrier generation, accumulation, and thermal and electrostrictive effects to the NLO response. This demonstrates that controlled defect engineering plays a decisive role in amplifying ultrafast NLO responses.

Overall, the 2 wt% Cu–TiO_2_ film shows the most pronounced NLA response, and the observed SA-RSA switching behavior highlights its strong potential for optical-limiting, mode-locking, and Q-switching applications. In contrast, the strongest SHG response exhibited by the 1 wt% Cu–TiO_2_ film highlights its suitability for frequency-doubling and wavelength-conversion applications, while the enhanced THG responses observed under picosecond excitation demonstrate the potential of Cu–TiO_2_ films for ultrafast photonic devices.

## 4 Conclusion

Pristine and Cu-doped TiO_2_ thin films (1–3 wt%) were successfully synthesized by spray pyrolysis technique and their structural, morphological, optical and NLO properties were systematically studied. XRD confirmed the anatase TiO_2_ phase, with the appearance of Cu-related diffraction peaks in the 2 and 3 wt% Cu–TiO_2_ films. The crystallite size increased from 23.27 to 30.28 nm upon Cu incorporation. The optical bandgap exhibited a blue shift from 3.26 to 3.40 eV upon Cu doping due to Burstein–Moss effect. The PL intensity increased with Cu doping, indicating enhanced radiative recombination in Cu–TiO_2_ thin films. XPS analysis confirmed successful Cu incorporation and revealed an increased oxygen vacancy concentration in the Cu-doped film. Nonlinear optical studies revealed an intensity-dependent SA-RSA transition in the 1 and 2 wt% Cu–TiO_2_ films. The 2 wt% Cu–TiO_2_ film exhibited the strongest nonlinear response, with the highest *β*_eff_ value of 5.90 × 10^−1^ m W^−1^ and Im*χ*^(3)^ value of 8.14 × 10^−10^ m^2^ V^−2^, under CW laser excitation. Under picosecond laser excitation, the same film exhibited a maximum third-order nonlinear susceptibility (*χ*^(3)^) of 3.85 × 10^−21^ m^2^ V^−2^, confirming the significant enhancement of nonlinear optical performance at the optimum Cu doping concentration. Under nanosecond excitation, the maximum SHG signal was observed for the 1 wt% Cu–TiO_2_ film, whereas the pristine thin film exhibited the strongest THG response. Collectively, these results demonstrate a relation between Cu-driven modifications in structural and electronic properties and the enhancement of NLO behavior. The optimized Cu–TiO_2_ thin films therefore emerge as promising materials for next-generation photonic technologies, including optical limiting and ultrafast nonlinear devices. Further investigations could explore excitation under shorter pulse durations, particularly in the femtosecond regime, and translate these materials into scalable device architectures for next-generation nonlinear photonic technologies.

## Conflicts of interest

There are no conflicts to declare.

## Data Availability

The data sets supporting this article are not publicly available at the time of publication as they are not currently in a format suitable for broad distribution or reuse. However, the data can be provided by the authors upon reasonable request.

## References

[cit1] Zhang Y. X., Wang Y. H. (2017). Nonlinear optical properties of metal nanoparticles: A review. RSC Adv..

[cit2] Serhan M. *et al.*, Total iron measurement in human serum with a smartphone, AIChE Annu. Meet. Conf. Proc., 2019, vol. 2019, 10.1039/x0xx00000x

[cit3] He Y. (2024). *et al.*, Ultrafast All-Optical Modulation and Efficient Third-Harmonic Generation in Two-Dimensional Perovskite Heterostructures. ACS Photonics.

[cit4] Kalanoor B. S. (2016). *et al.*, Third-Order Optical Nonlinearities in Organometallic Methylammonium Lead Iodide Perovskite Thin Films. ACS Photonics.

[cit5] Zhou Y., Mahmood S., Engelberg D. L. (2023). urna l P re f. Curr. Opin. Electrochem..

[cit6] Zibod S. (2023). *et al.*, Strong Nonlinear Response in Crystalline Quartz at THz Frequencies. Adv. Opt. Mater..

[cit7] Myronchuk G., Kyrychenko M., Rakus P., Jedryka J., Taboukhat S. (2026). Rare-earth doping of AgGaGe 3 Se 8 : A pathway to multifunctional materials for tunable optoelectronics. Opt. Mater..

[cit8] Khan M. U. (2021). *et al.*, Exploration of Nonlinear Optical Properties of Triphenylamine-Dicyanovinylene Coexisting Donor-π-Acceptor Architecture by the Modification of π-Conjugated Linker. Front. Mater..

[cit9] Zhao Y. (2022). *et al.*, Optical Properties of New Third-Order Nonlinear Materials Modified by Click Chemistry. Molecules.

[cit10] Mathai J. (2023). *et al.*, Substantial effect of Cr doping on the third-order nonlinear optical properties of ZnO nanostructures. Opt. Mater..

[cit11] Kumar M., Perumbilavil S., Goel A., Philip R. (2021). Enhanced optical nonlinearity in β-MnO2 nanowire network decorated with Ag nanoparticles. Opt. Mater..

[cit12] Abed S., Bougharraf H., Bouchouit K., Sofiani Z., Derkowska-zielinska B., Aida M. S. (2015). Superlattices and Microstructures Influence of Bi doping on the electrical and optical properties of ZnO thin films. Superlattices Microstruct..

[cit13] Cunha R., Valverde J. V. P., De Boni L., Misoguti L., Mendonça C. R. (2025). Nonlinear Refraction and Absorption in Polymers Used for Femtosecond Direct Laser Writing. ACS Omega.

[cit14] Dandasena B. and Naik R., Nickel-doped VS 4 Nanostructures as a Promising Candidate for Nonlinear Optical Limiter Application, 2026, pp. 1453–1465, 10.1039/d5tc03306c

[cit15] Das S. , Supriya S., Dandasena B., Parida A., and Naik R., Nonlinear Optical Response of La-Doped VTe 2 Nanomaterials for Optical Limiter, 2025, 10.1021/acsanm.5c02828

[cit16] Kumar P. C. , Pattnaik S., and Kumar J., NLO Applications : an Experimental and, 2026, pp. 4608–4624, 10.1039/d5tc03782d

[cit17] Kompa A., Kekuda D., Murari M. S., Mohan Rao K. (2022). Defect induced enhanced catalytic activity of Lu doped titanium dioxide (TiO2) thin films. Surf. Interfaces.

[cit18] Farooq N. (2024). *et al.*, Recent trends of titania (TiO2) based materials: A review on synthetic approaches and potential applications. J. King Saud Univ., Sci..

[cit19] Armaković S. J., Savanović M. M., Armaković S. (2023). Titanium Dioxide as the Most Used Photocatalyst for Water Purification: An Overview. Catalysts.

[cit20] Yang H., Yang B., Chen W., Yang J. (2022). Preparation and Photocatalytic Activities of TiO2-Based Composite Catalysts. Catalysts.

[cit21] Katta V. S., Chappidi V. R., Raavi S. S. K. (2023). Plasmonic Au NPs embedded Ytterbium-doped TiO2 nanocomposites photoanodes for efficient indoor photovoltaic devices. Appl. Surf. Sci..

[cit22] Cao S., Sui N., Zhang P., Zhou T., Tu J., Zhang T. (2022). TiO2 nanostructures with different crystal phases for sensitive acetone gas sensors. J. Colloid Interface Sci..

[cit23] Wu X., Shen C., Zhang X., Wang Y., jun Liu C. (2025). Dielectric surface engineering *via* TiO_2_ coating for enhanced plasma-driven efficient CO_2_ decomposition. Chem. Eng. J..

[cit24] Praveen P., Viruthagiri G., Mugundan S., Shanmugam N. (2014). Sol-gel synthesis and characterization of pure and manganese doped TiO_2_ nanoparticles - A new NLO active material. Spectrochim. Acta – Part A Mol. Biomol. Spectrosc..

[cit25] Khlyustova A., Sirotkin N., Kusova T., Kraev A., Titov V., Agafonov A. (2020). Doped TiO2: The effect of doping elements on photocatalytic activity. Mater. Adv..

[cit26] Ghamarpoor R., Fallah A., Jamshidi M. (2024). A Review of Synthesis Methods, Modifications, and Mechanisms of ZnO/TiO2-Based Photocatalysts for Photodegradation of Contaminants. ACS Omega.

[cit27] Tian J., Gao H., Kong H., Yang P., Zhang W., Chu J. (2013). Influence of transition metal doping on the structural, optical, and magnetic properties of TiO2 films deposited on Si substrates by a sol-gel process. Nanoscale Res. Lett..

[cit28] Liyanaarachchi H., Thambiliyagodage C., Liyanaarachchi C. (2023). Efficient photocatalysis of Cu doped TiO 2/g-C 3 N 4 for the photodegradation of methylene blue. Arab. J. Chem..

[cit29] Sahu M., Biswas P. (2011). Single-step processing of copper-doped titania nanomaterials in a flame aerosol reactor. Nanoscale Res. Lett..

[cit30] Choudhury B. , Dey M., and Choudhury A., Defect Generation, d–d Transition, and Band Gap Reduction in Cu-Doped TiO_2_ Nanoparticles, 2013, pp. 2–9

[cit31] Roy S., Tripathy N., Pradhan D., Sahu P. K., Kar J. P. (2018). Applied Surface Science Electrical characteristics of dip coated TiO 2 thin films with various withdrawal speeds for resistive switching applications. Appl. Surf. Sci..

[cit32] Priyalakshmi K., Goswami P., Chaturvedi H. (2022). Applied Surface Science Fabrication of nanocrystalline TiO 2 thin films using Sol-Gel spin coating technology and investigation of its structural , morphology and optical characteristics. Appl. Surf. Sci..

[cit33] Shabrina N. , Salsabila N. K., Sudarsono S., and Yudoyono G., Characterization of the structure, morphology, and optical properties of titanium dioxide thin film deposited by spray pyrolysis technique for self-cleaning glass, Journal of Physics: Conference Series, Institute of Physics, 2024, 10.1088/1742-6596/2780/1/012020

[cit34] Alotaibi A. M. et al. , Chemical Vapor Deposition of Photocatalytically Active Pure Brookite TiO 2 Thin Films, 2018, 10.1021/acs.chemmater.7b04944

[cit35] Kumi-Barimah E., Penhale-Jones R., Salimian A., Upadhyaya H., Hasnath A., Jose G. (2020). Phase evolution, morphological, optical and electrical properties of femtosecond pulsed laser deposited TiO2 thin films. Sci. Rep..

[cit36] Belachew A., Setia H., Shih S. (2023). Journal of Analytical and Applied Pyrolysis An comprehensive review on the spray pyrolysis technique : Historical context , operational factors , classifications , and product applications. J. Anal. Appl. Pyrolysis.

[cit37] Farzaneh A., Javidani M., Esrafili M. D., Mermer O. (2022). Optical and photocatalytic characteristics of Al and Cu doped TiO2: Experimental assessments and DFT calculations. J. Phys. Chem. Solids.

[cit38] Rajabi M., Abrinaei F. (2019). High nonlinear optical response of Lanthanum-doped TiO2 nanorod arrays under pulsed laser irradiation at 532 nm. Opt Laser. Technol..

[cit39] Raguram T., Rajni K. S. (2022). Synthesis and characterisation of Cu - Doped TiO2 nanoparticles for DSSC and photocatalytic applications. Int. J. Hydrogen Energy.

[cit40] Khan M. A. M., Nain P., Ahmed J., Ahamed M., Kumar S. (2022). Characterization and photocatalytic performance of hydrothermally synthesized Cu-doped TiO2 NPs. Opt. Mater..

[cit41] Zakir O. (2025). *et al.*, A study on the influence of metal Ag, Cu, and Fe doping on the morphological, structural, and photocatalytic activity of TiO2 nanostructures. J. Alloys Compd..

[cit42] Bensouici F. (2017). *et al.*, Optical, structural and photocatalysis properties of Cu-doped TiO 2 thin films. Appl. Surf. Sci..

[cit43] Byrne C., Moran L., Hermosilla D., Merayo N., Blanco Á., Rhatigan S., Hinder S., Ganguly P., Nolan M., Pillai S. C. (2019). Effect of Cu doping on the anatase-to-rutile phase transition in TiO2 photocatalysts: Theory and experiments. Appl. Catal., B.

[cit44] Ahmed S. A. (2017). Structural, optical, and magnetic properties of Cu-doped TiO_2_ samples. Cryst. Res. Technol..

[cit45] Krishnakumar V., Boobas S., Jayaprakash J., Rajaboopathi M., Han B., Louhi-Kultanen M. (2016). Effect of Cu doping on TiO2 nanoparticles and its photocatalytic activity under visible light. J. Mater. Sci.: Mater. Electron..

[cit46] Navyashree B. (2026). *et al.*, Precursor Concentration–Driven Modulation of harmonic generation responses in TiO2 nanostructures for sustainable photonic devices. Opt Laser. Technol..

[cit47] Morgan P. E. D., Partin D. E., Chamberland B. L., O'Keeffe M. (1996). Synthesis of paramelaconite: Cu4O3. J. Solid State Chem..

[cit48] Kolawole M. I., Hall T. A., Paudel J. (2026). Thermoelectrochemical Synthesis of Nanostructured Cupric Oxide (CuO) Using KMnO4as an Oxidant. ACS Omega.

[cit49] Das M., Bittencourt C., Pireaux J. J., Shivashankar S. A. (2012). Completely random nanoporous Cu 4O 3-CuO-C composite thin films for potential application as multiple channel photonic band gap based filter in the telecommunication wavelengths. Appl. Phys. A: Mater. Sci. Process..

[cit50] Ikram M. (2020). *et al.*, Dye degradation performance, bactericidal behavior and molecular docking analysis of Cu-doped TiO2nanoparticles. RSC Adv..

[cit51] Mingmongkol Y. (2022). *et al.*, Enhanced Photocatalytic and Photokilling Activities of Cu-Doped TiO2 Nanoparticles. Nanomaterials.

[cit52] Patle L. B., Labhane P. K., Huse V. R., Chaudhari A. L. (2015). Structural Analysis of Cu Doped TiO 2 Nanoparticles using Williamson-Hall Method. Int. J. Sci. Res. Sci. Eng. Technol..

[cit53] Saleem S. (2025). *et al.*, A comparative analysis of optical and electrical properties of pure CuO and Zn doped CuO nanoparticles for optoelectronic device applications. J. Sol-Gel Sci. Technol..

[cit54] Mehmood A. (2024). *et al.*, Innovative approach to synthesize Mo-Doped CuO Nanostructures: Uncovering structural and photocatalytic insights. J. Mol. Liq..

[cit55] Ahmadiasl R., Moussavi G., Shekoohiyan S., Razavian F. (2022). Synthesis of Cu-Doped TiO2 Nanocatalyst for the Enhanced Photocatalytic Degradation and Mineralization of Gabapentin under UVA/LED Irradiation: Characterization and Photocatalytic Activity. Catalysts.

[cit56] Mali D. G., Patil S. P., Sonawane G. H., Bhadane B. S., Mahajan V. K. (2025). Synthesis of TiO2 and Ag/Cu doped TiO2 nanoparticles in green approach and assess their structural, morphological, and photocatalytic applications. Hybrid Adv..

[cit57] Chandra Paul T., Podder J., Paik L. (2022). Effect of Fe doping on the microstructure, optical and dispersion energy characteristics of TiO2 thin films prepared *via* spray pyrolysis technique. Results Opt..

[cit58] Navas J. (2014). *et al.*, Experimental and theoretical study of the electronic properties of Cu-doped anatase TiO2. Phys. Chem. Chem. Phys..

[cit59] Pappas D. K., Boningari T., Boolchand P., Smirniotis P. G. (2016). Novel manganese oxide confined interweaved titania nanotubes for the lowerature Selective Catalytic Reduction (SCR) of NOx by NH3. J. Catal..

[cit60] Cuadra J. G. (2023). *et al.*, Applied Surface Science Multifunctional silver-coated transparent TiO 2 thin films for photocatalytic and antimicrobial applications. Appl. Surf. Sci..

[cit61] Aljuaid J., Timoumi A., Alamri S. N. (2022). Optical Materials : X Investigation of the impact of iron amounts on optical and physical properties of coated TiO 2 thin films used for PV solar cells. Opt. Mater. X.

[cit62] Karunagaran B., Rajendra Kumar R. T., Senthil Kumar V., Mangalaraj D., Narayandass S. K., Mohan Rao G. (2003). Structural characterization of DC magnetron-sputtered TiO2 thin films using XRD and Raman scattering studies. Mater. Sci. Semicond. Process..

[cit63] Taudul B. and Tielens F., On the Origin of Raman Activity in Anatase TiO_2_ (Nano) Materials: an *Ab Initio* Investigation of Surface and Size Effects, 2023, vol. 2 10.3390/nano13121856 PMC10301693 37368286

[cit64] Podelinska A. (2025). *et al.*, Structural and Spectroscopic Characterization of TiO2 Nanocrystalline Materials Synthesized by Different Methods. Nanomaterials.

[cit65] Albaidani K., Timoumi A., Belhadj W., Alamri S. N., Ahmed S. A. (2023). Structural , electronic and optical characteristics of TiO 2 and Cu-TiO 2 thin films produced by sol-gel spin coating. Ceram. Int..

[cit66] Zhang J., Toe C. Y., Kumar P., Scott J., Amal R. (2023). Engineering defects in TiO2 for the simultaneous production of hydrogen and organic products. Appl. Catal., B.

[cit67] Rajni T. R. K. S. (2019). Synthesis and analysing the structural, optical, morphological, photocatalytic and magnetic properties of – TiO_2_ and doped (Ni and Cu) – TiO_2_ nanoparticles by sol–gel technique. Appl. Phys. A.

[cit68] Saviot L. (2014). *et al.*, Optical and acoustic vibrations confined in anatase TiO2 nanoparticles under high-pressure. J. Phys. Chem. C.

[cit69] Jlaili M. (2025). *et al.*, Spray-Deposited TiO 2 –CuO Heterostructured Thin Films for Rifampicin Degradation and Solar Cell Application. ACS Omega.

[cit70] Göde F., Çelik A. (2024). The Structural and Optical Properties of Polycrystalline Copper Oxide Thin Films Synthesized Using the SILAR Technique. Karadeniz Fen Bilim. Derg..

[cit71] Giri S. and Kumar P. C., Enhanced Photodetectivity and Responsivity in In10Se70Te15Bi5 Film by Time-dependent Laser Irradiation for Photodetector Applications, 2025, pp. 46821–46837, 10.1039/d5ra06469d PMC12659727 41323717

[cit72] Gençyilmaz O. (2021). Transition metals (Cu/Co/Mn) doped NiO films: Structural, optical and morphological properties. J. Mater. Electron. Devices.

[cit73] Munir S., Shah S. M., Hussain H., Ali khan R. (2016). Effect of carrier concentration on the optical band gap of TiO2 nanoparticles. Mater. Des..

[cit74] Ganjoo A., Golovchak R. (2008). Computer program PARAV for calculating optical constants of thin films and bulk materials: Case study of amorphous semiconductors. J. Optoelectron. Adv. Mater..

[cit75] Naffouti W., Ben Nasr T., Briot O., Kamoun-Turki N. (2015). Effect of Sprayed Solution Volume on Physical Properties of Titanium Dioxide Thin Films. J. Electron. Mater..

[cit76] Mathew S. (2012). *et al.*, UV-visible photoluminescence of TiO2 nanoparticles prepared by hydrothermal method. J. Fluoresc..

[cit77] Nair R. V., Gayathri P. K., Gummaluri V. S., Nambissan P. M. G., Vijayan C. (2018). Large bandgap narrowing in rutile TiO2 aimed towards visible light applications and its correlation with vacancy-type defects history and transformation. J. Phys. D: Appl. Phys..

[cit78] Sahoo S., Surbhi K., Bhakta S., Das R., Sahoo P. K. (2024). Influence of defects on the linear and nonlinear optical properties of Cu-doped rutile TiO2 microflowers. Phys. Chem. Chem. Phys..

[cit79] Seetharaman A., Sivasubramanian D., Gandhiraj V., Soma V. R. (2017). Tunable Nanosecond and Femtosecond Nonlinear Optical Properties of C-N-S-Doped TiO2 Nanoparticles. J. Phys. Chem. C.

[cit80] Paul K. K., Jana S., Giri P. K. (2018). Tunable and High Photoluminescence Quantum Yield from Self-Decorated TiO2 Quantum Dots on Fluorine Doped Mesoporous TiO2 Flowers by Rapid Thermal Annealing. Part. Part. Syst. Charact..

[cit81] Preethi L. K., Antony R. P., Mathews T., Walczak L., Gopinath C. S. (2017). A Study on Doped Heterojunctions in TiO 2 Nanotubes : An Efficient Photocatalyst for Solar Water Splitting. Sci. Rep..

[cit82] Jin C., Liu B., Lei Z., Sun J. (2015). Structure and photoluminescence of the TiO2 films grown by atomic layer deposition using tetrakis-dimethylamino titanium and ozone. Nanoscale Res. Lett..

[cit83] Mondal S., Basak D. (2016). Defect controlled tuning of the ratio of ultraviolet to visible light emission in TiO2 thin films. J. Lumin..

[cit84] You Y. F. (2014). *et al.*, Structural characterization and optical property of TiO2 powders prepared by the sol-gel method. Ceram. Int..

[cit85] K S. (2017). *et al.*, Annealing Temperature Effect on the Physical Properties of Titanium Oxide Thin Films Prepared by the Sol-Gel Method. J. Phys. Chem. Biophys..

[cit86] Khan M. (2023). *et al.*, Investigation of Photoluminescence and Optoelectronics Properties of Transition Metal-Doped ZnO Thin Films. Molecules.

[cit87] Qahtan T. F., Owolabi T. O., Saleh T. A. (2024). X-ray photoelectron spectroscopy of surface-treated TiO2 mesoporous film by 500 eV argon ion beam. J. Mol. Liq..

[cit88] Wu K., Shi Z., Wang X., Wang J. (2022). Effect of Ce-Doping on Microstructure and Adsorption- Photodegradation Behaviors of the Hydrothermally-Synthesized TiO2 Nanotubes. Crystals.

[cit89] Mikaeili F., Rahaman M. M., Gouma P. I. (2025). 3D Self-Supported Visible Light Photochemical Nanocatalysts. Adv. Sci..

[cit90] Z-derivatized, B. S. C. T. Nanostructures , Photocatalytic and Electrocatalytic Properties of Cu-Loaded, 2021, pp. 1–19 10.3390/nano11081904 PMC8399894 34443738

[cit91] Krishnan P. (2017). *et al.*, Characterization of photocatalytic TiO2 powder under varied environments using near ambient pressure X-ray photoelectron spectroscopy. Sci. Rep..

[cit92] Akshay V. R. (2018). *et al.*, Defect mediated mechanism in undoped, Cu and Zn-doped TiO 2 nanocrystals for tailoring the band gap and magnetic properties. RSC Adv..

[cit93] Xiao B. (2023). *et al.*, Cu surface doped TiO2: Constructing Cu single-atoms active sites and broadening the photo-response range for efficient photocatalytic hydrogen production. Chem. Eng. J..

[cit94] Xu J., Zheng Y., Tian J., Zhao Y., Zheng H. (2024). Enhanced desulfurization performance of model fuel by Cu-ZnO/TiO2 heterostructure. RSC Adv..

[cit95] Sheik-Bahae M., Said A. A., Wei T. H., Hagan D. J., Van Stryland E. W. (1990). Sensitive Measurement of Optical Nonlinearities Using a Single Beam. IEEE J. Quantum Electron..

[cit96] Stavrou M. (2023). *et al.*, Exceptional ultrafast nonlinear optical response of functionalized silicon nanosheets. Nanoscale.

[cit97] Ariyamuthu R., G M., Arumugam N., Almansour A. I., Sudarshan K., S J. (2025). Bio-mediated green synthesis of copper oxide nanoparticles using plant extract and its applications to optical switching in nonlinear optics. Part. Sci. Technol..

[cit98] Pooja Shaji R. (2025). *et al.*, Influence of cobalt doping on the structural and third order nonlinear optical properties of MgO nanostructures. Mater. Sci. Eng., B.

[cit99] Endale G., Mohan D. (2024). Analysis of *z*-scan signals for thin and thick optical materials through strong focusing with enhanced sensitivity. J. Opt..

[cit100] Arun K. J., Jayalekshmi S. (2015). Third order nonlinear optical constants of L-alaninium oxalate. Photonics Lett. Pol..

[cit101] Khan P., Yadav R. K., Mondal A., Rout C. S., Adarsh K. V. (2021). Pulse-duration dependence of saturable and reverse saturable absorption in ZnCo2O4 microflowers. Opt. Mater. (Amst)..

[cit102] Furey B. J. , Barba-barba R. M., Carriles R., Bernal A., Mendoza B. S., and Downer M. C., Im {χ(3)} Spectra of 110-cut GaAs, GaP, and Si Near the Two-Photon Absorption Band Edge 1, 2021

[cit103] Romphosri S., Sin P., Pornpawee O., Rakchart K., Tanant T. (2022). Two - photon absorption cross - section investigation of visible - light photoinitiators under Q - switched Nd : YAG nanosecond pulse laser at 1064 nm. Appl. Phys. B.

[cit104] Jia J. , Li J., Zhang T., Lu Y., and Song Y., Monomeric and Dimeric Structures, 2024, pp. 11064–11072, 10.1039/d3cp06059d 38529570

[cit105] Ding M. and Chen D., 9 - Lanthanide Ions Doped Upconversion Nanomaterials: Synthesis, Surface Engineering, and Application in Drug Delivery, Elsevier Inc., 2016, 10.1016/B978-0-323-47347-7/00009-4

[cit106] Thomas S. S., Joe I. H. (2024). Intensity-dependent SA-RSA switching in nickel-chromium transition metal oxide nanoparticles. Opt. Mater..

[cit107] Gao L. (2022). *et al.*, Nonlinear Optical Properties of Pyrene Derivatives Based on a Donor-Acceptor Structure and Its Polyurethane Composites. ACS Omega.

[cit108] Elmali A. , Karatay A., and Donar Y. O., Controlling the Nonlinear Absorption Characteristics of TiO_2_/carbon Nanocomposites on Films, 2018, 108, pp. 510–514, 10.1016/j.optlastec.2018.07.028

[cit109] Tantray F. A., Agrawal A., Gupta M., Andrews J. T., Sen P. (2016). Effect of oxygen partial pressure on the structural and optical properties of ion beam sputtered TiO 2 thin films. Thin Solid Films.

[cit110] Jena T. (2024). *et al.*, Nonlinear optical studies of Bismuth-doped Titanium di-oxide colloids achieved by femtosecond *Z*-Scan technique. Opt. Mater..

[cit111] Twinkle J. (2024). *et al.*, Strong Two and Three-Photon Absorption Coefficients of Gold-Doped Titanium Di-Oxide Nanoparticles Achieved by Femtosecond *Z*-Scan Technique. Plasmonics.

[cit112] Abdel Samad F. (2024). *et al.*, Experimental Investigation of the Optical Nonlinearity of Laser-Ablated Titanium Dioxide Nanoparticles Using Femtosecond Laser Light Pulses. Nanomaterials.

[cit113] Asaldoust F., Mabhouti K., Jafari A., Taleb-abbasi M. (2025). Study of *Z*-scan technique, dispersion energy, and Wemple – DiDomenico model on Cu, Cr and Fe doped NiO nanopowder for the determination of nonlinear and linear optical characteristics. Sci. Rep..

[cit114] Nagaraja K. K., Pramodini S., Kumar A. S., Nagaraja H. S., Poornesh P., Kekuda D. (2013). Third-order nonlinear optical properties of Mn doped ZnO thin films under cw laser illumination. Opt. Mater..

[cit115] Yi G. (2017). *et al.*, Nonlinear third harmonic generation at crystalline sapphires. Opt. Express.

[cit116] Liu T., Xiao S., Li B., Gu M., Luan H., Fang X. (2022). Third- and Second-Harmonic Generation in All-Dielectric Nanostructures: A Mini Review. Front. Nanotechnol..

[cit117] Bowman A. R. , Boroviks S., Karaman O. C., Herz L. M., Olivier J. F., and Tagliabue G., Plasmon Induced Delocalized Second-Harmonic Generation towards Buried-Interface Spectroscopy, arXiv, 2026, preprint, arXiv:2605.00575, 10.48550/arXiv.2605.00575

[cit118] Nakamura S. et al. , Optical Second-Harmonic Generation from the Anatase TiO (101) Face, 2000, vol. 89, pp. 862–864

[cit119] Manuscript A. , Collective Nonlinear Electric Polarization *via* Defect-Driven, 2022

[cit120] Li K. , Lin J., Zhang Z., Sato R., Shimizu H., and Matsukuma H., Applied Sciences Investigation of Angle Measurement Based on Direct Third Harmonic Generation in Centrosymmetric Crystals, p. 2023

[cit121] Otieno C. O. , Second-Order Non Linear Optical Properties of Zinc Oxide and Aluminum doped Zinc Oxide Thin Films grown by Atomic Layer deposition, arXiv, 2023, preprint, arXiv:2304.14234, 10.48550/arXiv.2304.14234, https://arxiv.org/pdf/2304.14234

[cit122] Kubodera K., Kobayashi H. (1990). Determination of Third-Order Nonlinear Optical Susceptibilities for Organic Materials by Third-Harmonic Generation. Mol. Cryst. Liq. Cryst. Inc. Nonlinear Opt..

[cit123] Ben Yahya S., El Karout H., Sahraoui B., Barillé R., Louati B. (2024). Innovative synthesis, structural characteristics, linear and nonlinear optical properties, and optoelectric parameters of newly developed A2ZnGeO4 (A = K, Li) thin films. RSC Adv..

[cit124] Chtouki T. (2017). *et al.*, Spin-coated nickel doped cadmium sulfide thin films for third harmonic generation applications. J. Alloys Compd..

[cit125] Chtouki T. (2017). *et al.*, Comparison of structural, morphological, linear and nonlinear optical properties of NiO thin films elaborated by Spin-Coating and Spray Pyrolysis. Optik.

[cit126] Abed S. (2025). *et al.*, Zinc doping-induced modulation of optical and nonlinear optical properties in MgO thin films deposited by dip coating. Opt. Quantum Electron..

[cit127] Mathew S. (2018). Cu-Doped TiO_2_: Visible Light Assisted Photocatalytic Antimicrobial Activity. Appl. Sci..

[cit128] Seetharaman A. , Sivasubramanian D., Gandhiraj V., and Soma V. R., Tunable Nanosecond and Femtosecond Nonlinear Optical Properties of C–N– S-Doped TiO_2_ Nanoparticles, 2017, pp. 24192–24205, 10.1021/acs.jpcc.7b08778

